# Metabolomic Analysis of Defense-Related Reprogramming in *Sorghum bicolor* in Response to *Colletotrichum sublineolum* Infection Reveals a Functional Metabolic Web of Phenylpropanoid and Flavonoid Pathways

**DOI:** 10.3389/fpls.2018.01840

**Published:** 2019-01-04

**Authors:** Fidele Tugizimana, Arnaud T. Djami-Tchatchou, Paul A. Steenkamp, Lizelle A. Piater, Ian A. Dubery

**Affiliations:** Department of Biochemistry, Research Centre for Plant Metabolomics, University of Johannesburg, Johannesburg, South Africa

**Keywords:** *Colletotrichum sublineolum*, 3-deoxyanthocyanidin, metabolomics, phenylpropanoid, flavonoid, phytoalexins, *Sorghum bicolor*

## Abstract

The metabolome of a biological system provides a functional readout of the cellular state, thus serving as direct signatures of biochemical events that define the dynamic equilibrium of metabolism and the correlated phenotype. Hence, to elucidate biochemical processes involved in sorghum responses to fungal infection, a liquid chromatography-mass spectrometry-based untargeted metabolomic study was designed. Metabolic alterations of three sorghum cultivars responding to *Colletotrichum sublineolum*, were investigated. At the 4-leaf growth stage, the plants were inoculated with fungal spore suspensions and the infection monitored over time: 0, 3, 5, 7, and 9 days post inoculation. Non-infected plants were used as negative controls. The metabolite composition of aqueous-methanol extracts were analyzed on an ultra-high performance liquid chromatography system coupled to high-definition mass spectrometry. The acquired multidimensional data were processed to create data matrices for multivariate statistical analysis and chemometric modeling. The computed chemometric models indicated time- and cultivar-related metabolic changes that reflect sorghum responses to the fungal infection. Metabolic pathway and correlation-based network analyses revealed that this multi-component defense response is characterized by a functional metabolic web, containing defense-related molecular cues to counterattack the pathogen invasion. Components of this network are metabolites from a range of interconnected metabolic pathways with the phenylpropanoid and flavonoid pathways being the central hub of the web. One of the key features of this altered metabolism was the accumulation of an array of phenolic compounds, particularly *de novo* biosynthesis of the antifungal 3-deoxyanthocynidin phytoalexins, apigeninidin, luteolinidin, and related conjugates. The metabolic results were complemented by qRT-PCR gene expression analyses that showed upregulation of defense-related marker genes. Unraveling key characteristics of the biochemical mechanism underlying sorghum—*C. sublineolum* interactions, provided valuable insights with potential applications in breeding crop plants with enhanced disease resistance. Furthermore, the study contributes to ongoing efforts toward a comprehensive understanding of the regulation and reprogramming of plant metabolism under biotic stress.

## Introduction

Sorghum [*Sorghum bicolor* (L.) Moench], is a major cereal food crop in many parts of the world, particularly in Africa and Asia, and positioned as the fifth most valuable and highly produced cereal crop worldwide (Althwab et al., 2015). It plays an important role in sustainable grain production and food security, particularly in semi-arid and tropic areas (Dicko et al., 2006). Although sorghum is used primarily as a food crop in Africa, Asia, and Latin America, it is mainly utilized for animal livestock feed and bioenergy generation in Australia and the United States of America (Poloni and Schirawski, 2014; Wu et al., 2017). Compared to other grain crops, sorghum has high levels of phenolic compounds which possess antioxidant properties and other biological activities that can benefit human health (Awika and Rooney, 2004). Phenolic compounds are the most widely distributed secondary metabolites in plants and the major classes of this family of secondary metabolites are phenolic acids, flavonoids, and tannins (Quideau et al., 2011; Cheynier et al., 2013). The phenolic compounds found in sorghum include phenolic acids and flavonoids. The phenolic acids are mainly derivatives of cinnamic—and benzoic acids; whereas the flavonoids may include flavanols, flavones, flavanones, flavonones, anthocyanins, and derivatives (Quideau et al., 2011). The profiles and levels of these phenolic compounds in sorghum are dependent on the genotype as well as growth—and environmental conditions. Increasing awareness of the health benefits of these phytochemicals has redefined the value of sorghum as a most nutritionally valuable cereal crop worldwide (Awika and Rooney, 2004). For these reasons, there is renewed interest in sorghum research for a detailed and extended description of its phytochemical composition, and for in-depth understanding of the cellular and organismal biochemistry of sorghum in adverse environments (Balmer et al., 2013; Althwab et al., 2015).

Like all plants, sorghum is constantly prone to attacks by a plethora of potential pathogens and other biotic stressors, which can lead to severe yield losses. One of the biotic stressors that poses a great threat to sorghum production is the hemibiotrophic fungus, *Colletotrichum sublineolum*. It is the causal agent of a destructive above-ground disease, anthracnose, which can lead to 70% yield loss under severe epidemics due to defoliation and tissue death (Basavaraju et al., 2009; Balmer et al., 2013). The pathogen, *C. sublineolum*, is capable of destructively infecting all aerial tissue of sorghum plants, especially leaf tissue, impacting negatively on the grain yield and quality. The symptoms and severity of the post-infection diseased state on sorghum vary depending on the interaction between host plant, the environment and variation in virulence within the pathogen population. Some of the symptoms include elongated lesions which coalesce as the disease progresses to cover most of the leaf tissue, and few or numerous fungal fruiting bodies (acervuli) visible as black spots at the center of the leaf lesion as the fungus sporulates (Tesso et al., 2012). The control of the disease through the development of resistant cultivars has been difficult and often less successful, even in regions with endemic anthracnose, due to the hypervariable nature of *C. sublineolum*. In addition, environmental conditions also effects disease development. Development of sorghum anthracnose is accompanied and regulated by different cellular reprogramming events in both *C. sublineolum* and sorghum plants; and understanding cellular and molecular responses of both the pathogen and the host during the infection process could provide informative insights, leading to sustainable application avenues in combating the fungal infection of sorghum crops.

Although some key molecular events that characterize the sorghum defense responses to *C. sublineolum* infection have been described (Supplementary Information; Dicko et al., 2005; Anjum et al., 2013), the mechanistic physiological and molecular bases that determine the outcome of this phytopathogenic interaction are still not fully elucidated (Basavaraju et al., 2009; Tesso et al., 2012; Vargas et al., 2012; McDowell, 2013). Hence, unraveling the intricacies of the molecular mechanisms underlying the sorghum defensive responses to the fungal infection could provide descriptive insights, which along with existing biochemical knowledge, can be explored for designing and development of improved sorghum cultivars. Thus, this metabolomics-based investigation reports on the characterization of multi-parametric metabolic reprogramming that underlies the induced defense mechanisms in sorghum plants responding to *C. sublineolum* infection.

## Materials and Methods

### Sorghum Plant Preparation

Sorghum [*Sorghum bicolor* (L.) Moench] seeds of three South African cultivars were used, namely Amazi Mhlophe (abbreviated as MHL or M), NS 5511 (referred to as bitter and abbreviated as BTT or B) and NS 5655 (referred to as sweet, abbreviated as SWT or S; Agricol, Pretoria, South Africa). NS 5511 and NS 5655 are both grain sorghum hybrids of the malting class. NS 5511 is a red, tannin type, classified as GH (high levels of condensed tannin, responsible for dark testa), while NS 5655 is classified as GM (no condensed tannins, no dark testa). Both NS 5511 and NS 5655 have a rating of “3” (on a 1–9 scale with 1 being the most resistant) in terms of exhibiting disease resistance against head smut, leaf disease and root rot (Capstone Seeds, Howick, South Africa). In contrast, Amazi-Mhlophe is an open pollinated, white grain sorghum type. The three cultivars are listed in the variety list of the Registrar of Plant Improvement, Department of Agriculture, Forestry and Fisheries, South Africa (www.daff.gov.za/daffweb3/Branches/Agricultural-Production-Health-Food-Safety/Plant-Production/Verietal-Listing).

Seeds were surface-sterilized in a 1.2% sodium hypochlorite solution and rinsed with sterile water before being placed in glass Petri dishes with soaked paper towel and incubated at 28°C in the dark for 48 h to germinate. The seedlings were then planted in horticultural-grade vermiculite under fluorescent lights with a 12 h light (≈85 μmol m^−2^ s^−1^) and 12 h dark cycle. The temperature was kept at 22–27°C. The seedlings were watered (tap water with Multisol N fertilizer, Culterra, Muldersdrift, South Africa) regularly. The study was designed to monitor the plant response to fungal infection over time for 1, 3, 5, 7, and 9 days post infection (d.p.i.) as based on initial optimisation studies. The seedlings were planted in replicas of at least 10 plants per time point, and all plants were grown at the same time under the same environmental conditions. The complete experimental design included three biological repeats.

### Preparation of *Colletotrichum sublineolum* Spore Suspensions

A pathogenic isolate of *C. sublineolum* (PPRI 7183) from fodder, grown, and maintained on potato dextrose agar (PDA), was obtained from the National Collection of Fungi, Plant Protection Institute, Agricultural Research Council (ARC), Pretoria, South Africa. The working sub-cultures were maintained on half-strength PDA solid media in Petri dishes. For spore production, the fungus was sub-cultured into 20% aqueous V8 medium (pH 3.9), which was prepared by mixing 100% V8 vegetable juice (Campbell Soup Company Camden, NJ, USA) and distilled water (1:5, v/v), and autoclaved. One hundred milli liter of the autoclaved media in 250 mL Erlenmeyer flasks were then inoculated with fungal mycelia (5 mycelia plugs per flask) from *C. sublineolum* cultures growing on half-strength PDA plates. The inoculated aqueous V8 media was incubated with constant shaking at 130 rpm, 12 h light cycle, for 7 d. The flask cultures were harvested after 7 d of growth by filtering the medium under vacuum through muslin cloth to remove the mycelial clumps. The spores present in the filtrate were pelleted by centrifugation at 5,000 × *g* for 15 min, washed by suspension (using autoclaved distilled water) and centrifugation, and diluted to the required concentration. The spore concentration was determined using a haemocytometer and light microscope at 400 × magnification, and adjusted to 10^6^ spores mL^−1^.

### Inoculation of the Sorghum Seedlings

At the four-leaf growth stage (25 days after sowing), the sorghum plant leaves were treated by spraying with the fungal spore suspension, adjusted to 10^6^ spores mL^−1^, until run-off. The control plants were not sprayed. After inoculum application, the treated plants were incubated at 30°C in an incubator to provide 100% relative humidity, in darkness for 24 h. Following the 24 h incubation period, the plants were then exposed again to the same initial conditions: with cycles of 12 h fluorescence light (≈85 μmol m^−2^ s^−2^) and 12 h darkness, and the temperature kept at 22–27°C. Post-treatment harvesting of the plants was done for all cultivars at 1, 3, 5, 7, and 9 d.p.i. by cutting off the leaves and immediate storage at −80°C until metabolite extraction. Similarly, the non-treated (negative controls) plants were harvested at 1, 5, and 9 days.

### Metabolite Extraction and Sample Preparation

Metabolites were extracted from treated and non-treated plant leaves using 80% cold aqueous-methanol, in a ratio of 1:15 (w/v), at 4°C. The mixture was homogenized using an Ultra Turrax homogeniser, followed by sonication using a probe sonicator (Bandelin Sonopuls, Berlin, Germany) set at 55% power for 15 s, repeated 3 times. The homogenates were centrifuged at 5,000 × *g* for 10 min at 4°C. The supernatants were concentrated by evaporating to complete dryness and re-suspending the dried extracts in 300 μL 50% aqueous-methanol. The samples were then filtered through 0.22 μm nylon syringe filters into HPLC glass vials fitted with 500 μL inserts. The filtered extracts were kept at −20°C until analyzed. The methanol used was LC-grade (Romil Pure Chemistry, Cambridge, UK) and ultrapure water. The quality control (QC) samples were pooled samples prepared by pipetting and mixing aliquots of equal volume from all samples.

### Sample Analyses on an UHPLC-HDMS Analytical Platform

Ultra-high performance liquid chromatography coupled to high-definition mass spectrometry (UHPLC-MS) was performed on a Waters Acquity UHPLC coupled in tandem to a Waters photodiode array (PDA) detector and SYNAPT G1 Q-TOF mass spectrometer (Waters Corporation Milford, USA). Chromatographic separation of the aqueous-methanol extracts was done using a Waters HSS T3 C18 column (150 mm × 2.1 mm × 1.8 μm) thermostatted at 60°C. Although the T3 column is classified as a C18 reverse phase type, it is able to separate some polar compounds in addition to the non-polar compounds. Elution gradient was carried out with a binary solvent system consisting of 0.1% aqueous formic acid (solvent A) and 0.1% formic acid in acetonitrile (Romil Pure Chemistry, Cambridge, UK; solvent B) at a flow rate of 0.4 mL min^−1^. The initial conditions were 98% A and 2% B and held for 1 min. A gradient was applied to change the chromatographic conditions to 30% A and 70% B at 14 min; and changed to 5% A and 95% B at 15 min. These conditions were held for 2 min and then changed to the initial conditions at 18 min. The analytical column was allowed to calibrate for 2 min before the next injection. The total chromatographic run time was 20 min and the injection volume was 2 μL. Each sample was analyzed in triplicate to account for any analytical variability. Solvent blanks and the QC samples were also analyzed in parallel with the sample extracts (described below).

High definition mass spectrometry analyses were performed on a Waters SYNAPT G1 Q-TOF MS system in V-optics operated in both positive and negative electrospray ionization (ESI) modes. Leucine encephalin (50 pg mL^−1^), [M + H]^+^ = 55.2766 and [M–H]^−^ = 554.2615, was used as a reference calibrant, being continuously sampled every 15 s, producing an average intensity of 350 counts scan^−1^ in centroid mode. Using this reference, the MassLynx™ software automatically correct the centroid mass values in the sample for small deviations from the exact mass measurement, giving typical mass accuracies between 1 and 3 mDa. The capillary and sampling cone voltages were 2.5 kV and 30 V, respectively. The extraction cone was set at 4.0 V. The source temperature used was 120°C and the desolvation temperature 450°C; cone gas flow 50 L h^−1^ and desolvation gas flow of 550 L h^−1^. A scan time of 0.2 s was used with a 100–1,000 Da mass range. The nebulisation gas used was nitrogen with a 700 L h^−1^ flow rate. The data were acquired with different collision energies (MS^E^) 0–30 eV to obtain as much structural information as possible of the detected compounds. The software used to control the hyphenated system and perform all data manipulation was MassLynx™ 4.1 (SCN 704, Waters Corporation Milford, USA).

The QC (pooled) samples were used to condition the LC-MS analytical system so as to assess the reliability and reproducibility of the analysis, and for non-linear signal correction (Godzien et al., 2015; Broadhurst et al., 2018). Sample acquisition was randomized and the QC sample (6 injections) was analyzed every 10 injections to monitor and correct changes in the instrument response. Furthermore, 6 QC runs were performed at the beginning and end of the batch to ensure system equilibration. Such sample randomization provides stochastic stratification in sample acquisition so as to minimize measurement bias. In the principal component analysis (PCA) space, the QC samples were clustered closely to each other, confirming thus the stability of the LC-MS system used, the reliability and reproducibility of the analysis. The blank samples (50% aqueous methanol) were randomly run to monitor background noise.

### Data Analysis: Data Set Matrix Creation and Chemometric Analyses

The centroid-raw data obtained from UHPLC-HDMS (both ESI positive and—negative modes) were pre-processed (peak picking, noise filtering, retention time (Rt) alignment, peak integration, and normalization) using MassLynx XS™ 4.1 software (Waters Corporation, Manchester, UK). The MarkerLynx™ application manager of the MassLynx software was used for matrix creation, producing a matrix of (Rt-*m/z*) variable pairs, with *m/z* peak intensity for each sample. MarkerLynx software parameters were set to process the 1–15 min Rt range of the chromatograms and *m/z* domain of mass range 100–1,000 Da. The Rts were allowed to differ by ± 0.2 min and the *m/z* values by ± 0.05 Da. The mass tolerance used was 0.01 Da, and the intensity threshold was 100 counts. Only data matrices that had noise level <50% (MarkerLynx cut off) were retained for downstream chemometric and statistical analyses. After data pre-processing, the number of metabolite features (Rt, *m/z*) in the clean data sets were 1536 in ESI positive and 2759 in ESI negative data sets. These data matrices were exported into SIMCA (soft independent modeling of class analogy) software, version 14 (Umetrics, Umeå, Sweden) for statistical analyses. Two unsupervised methods, PCA and hierarchical clustering analysis (HCA), and a supervised modeling, orthogonal projection to latent structures-discriminant analysis (OPLS-DA), were employed. This included the OPLS-DA S plots and the Variable Importance in Projection (VIP) plots. These multivariate methods attempt to highlight trends and groupings within a data set, subsequently facilitating the understanding of the relationships between- and within the samples (Trygg et al., 2007; Tugizimana et al., 2013).

To ensure that the biological question of the study is accurately answered as competently as possible, data scrutiny was meticulously done following the data pre-processing steps. This included assessment of the number of extracted features (<10,000 features, as a rule of thumb), applying the 80% rule (i.e., features found in <20% of the analyzed samples were removed) and monitoring the quality of data and stability of the analysis using QC samples. Data transformation methods such as centering, scaling or transformation were exploratively employed to put all variables on equal footing, minimize variable redundancy and adjust for measurement errors (Tugizimana et al., 2016). A non-linear iterative partial least squares algorithm (in-built within SIMCA; Nelson et al., 1996) was used to manage the missing values, with a correction factor of 3.0 and a default threshold of 50%. A seven-fold cross-validation (CV) method (Bro et al., 2008) was applied as a tuning procedure in computing the chemometric models; and only the components positively contributing to increase the prediction ability of the model (*R1* significant components) were considered. Furthermore, thorough model validations were rigorously and consistently applied; and only statistically satisfactory models were examined and used in data mining for knowledge discovery. For PCA, the cumulative *R*^2^ (explained variation) and Q^2^ (predicted variation) of used models were higher than 0.5. Furthermore, for OPLS-DA models, the analysis of variance testing of cross-validated predictive residuals (CV-ANOVA), *p*-values were below 0.05. The specific values for these validation parameters (and others) are provided in the results section.

The study design information, LC-MS raw data, analyses and data processing information, and the metadata have been deposited to the EMBL-EBI metabolomics repository—MetaboLights database with the identifier (accession number) MTBLS791.

### Metabolite Identification, and Metabolic Pathway and Network Analyses

#### Metabolite Identification

For metabolite identification, the data matrices from MarkerLynx-based data processing were exported to the Taverna workbench (www.taverna.org.uk) for PUTMEDID_LCMS Metabolite ID workflows (Brown et al., 2011). The Taverna workflows allow for integrated, automated and high-throughput annotation and putative metabolite identification from LC-ESI-MS metabolomic data. The workflows consist of correlation analysis, metabolic feature annotation, and metabolite annotation. A data matrix from MarkerLynx-based data processing was firstly formatted to match the Taverna workbench requirements. Three main workflows formed the Taverna Metabolite ID procedure: (**i**) Pearson-based correlation analysis (*List_CorrData*), (**ii**) metabolic feature annotation (*annotate_Massmatch*)—allowing for grouping together ion peaks with similar features such as Rt, and annotating features with the type of *m/z* ion (molecular ion, isotope, adduct, others) believed to originate from the same compound. The elemental composition/molecular formula (MF) of each *m/z* ion was then automatically calculated; and (**iii**) metabolite annotation (*matchMF-MF*) of the calculated MF (from the output file from workflow 2) was automatically compared and matched to the MF from a pre-defined reference file of metabolites.

For confidence in metabolite annotation, the following steps were performed: (i) the calculated MF of a selected metabolite candidate was manually searched against databases and bioinformatics tools, mainly the Dictionary of Natural Products (DNP; www.dnp.chemnetbase.com), Chemspider (www.chemspider.com), PlantCyc (www.plantcyc.org), Knapsack database (http://kanaya.naist.jp/KNApSAcK/) and KEGG (www.genome.jp/kegg/); (ii) structural confirmation through careful inspection of fragmentation patterns by examining the MS^1^ and MS^E^ spectra of the selected metabolite candidate; (**iii**) comparative assessment with/against annotation details of metabolites in sorghum, reported in literature, particularly in Kang et al. (2016). Metabolites were annotated to level 2 as classified by the Metabolomics Standard Initiative (MSI; Sumner et al., 2007).

The presence and abundance of specific molecular features or identified metabolites (as expressed as the integrated peak areas in the data matrix X) was infographically captured by unsupervised color-coded-PCA scores plots using the SIMCA software.

#### Metabolic Pathway and—Network Analyses

Ingenuity pathway analysis (IPA) of metabolites identified/selected by OPLS-DA were performed with the MetPA (Metabolomics Pathway Analysis) component of the MetaboAnalyst bioinformatics tool suite (version 3.0; http://www.metaboanalyst.ca/), enabling the identification of the affected metabolic pathways, analysis thereof and visualization. IPA uses high-quality KEGG metabolic pathways as the supporting knowledge base. The identified significant metabolites (with respective KEGG identifiers, Table 1) were thus uploaded into MetPA tool for pathway analysis. The possible biological roles were evaluated by enrichment analysis. An over-representation approach, based on a hypergeometric test algorithm, was used for pathway enrichment analysis; and pathway topological analysis was based on relative betweenness centrality. Since many pathways are tested at the same time, both Holm-Bonferroni and false discovery rate procedures were used to adjust for the statistical *p*-values from enrichment analysis. Furthermore, correlation-network analyses were used to examine metabolite associations and interpret chemometric results within a comprehensive biological and experimental context. Thus, a biochemical and chemical similarity network was constructed between all OPLS-DA selected and annotated metabolites. MetaMapR (https://dgrapov.github.io/MetaMapR) was used to identify metabolic precursors to product relations based on KEGG identifiers (Grapov et al., 2015).

**Table 1:** Summary of annotated (MI-level 2) metabolites that contributed to the discriminating variability in the altered metabolomes as described by chemometric models.

	**Metabolites**	***m/z***	**Rt (min)**	**Ion/Adduct**	**ESI**	**MF**	**MW**	***p*-value**	**FC_B**	**FC_S**	**FC_M**	**Direction**
1	(-)-Jasmonic acid methyl ester	245.1149	1.98	M+Na	Neg	C_13_H_20_O_3_	224.30	0.0017	1.095	1.360	1.107	Increase
2	1,2-bis-O-Sinapoyl-beta-D-glucoside	591.1695	6.59	M-H	Neg	C_28_H_32_O_14_	592.55	0.0013	1.059	1.625	1.001	Increase
3	1-O-Sinapoyl-beta-D-glucose	385.1144	5.56	M-H	Neg	C_17_H_22_O_10_	386.35	0.0000	2.437	1.020	1.021	Increase
4	2-Coumarate	163.0396	4.51	M-H	Neg	C_9_H_8_O_3_	163.15	0.0484	1.604	0.798	1.978	Increase
5	3-Methyl-4-cis-hydroxy-2-butenal	121.0273	5.13	M-H_Na	Neg	C_5_H_8_O_2_	101.12	0.0000	1.004	0.879	1.334	Increase
6	4-Coumaroylshikimate	357.0381	3.26	M-K	Neg	C_16_H_16_O_7_	320.30	0.0038	0.767	1.248	1.142	Decrease
7	4-Hydroxycoumarin	163.0385	4.69	M+H	Pos	C_9_H_6_O_3_	162.14	0.0408	3.830	2.957	0.465	Increase
8	6-Aminohexanoate	170.0586	4.11	M+H_K	Pos	C_6_H_13_NO_2_	131.18	0.0076	7.432	6.021	5.703	Increase
9	Abscisate	355.1131	4.02	M+H_FANa	Pos	C_15_H_20_O_4_	264.32	0.0000	3.912	3.814	2.933	Increase
10	Apigenin	271.0620	6.07	M+H	Pos	C_15_H_10_O_5_	270.24	0.0288	35.023	17.605	6.901	Increase
11	Apigenin 7-O-neohesperidoside	577.1548	5.95	M-H	Neg	C_27_H_30_O_14_	578.52	0.0016	21.230	11.350	3.234	Increase
12	Apigenin 7-O-β-D-glucoside	431.0978	6.67	M-H	Neg	C_21_H_20_O_10_	432.38	0.0008	11.770	10.853	2.538	Increase
13	Apigeninidin	255.0481	6.15	M+H	Pos	C_15_H_11_${\text{O}}_{4}^{+}$	255.24	0.0000	38.975	22.027	20.551	Increase
14	Caffeoylglucarate	371.0625	4.01	M-H	Neg	C_15_H_16_O_11_	372.28	0.0550	0.832	4.237	0.283	Decrease
15	Caffeoylquinate	353.0879	4.67	H-H	Neg	C_16_H_18_O_9_	354.31	0.0000	4.121	1.996	1.195	Increase
16	Coniferaldehyde glucoside	356.1344	3.26	M-NH3	Neg	C_16_H_20_O_8_	340.33	0.0000	0.651	0.748	0.200	Decrease
17	Coniferin	343.1373	4.75	M+H	Pos	C_16_H_22_O_8_	342.34	0.0000	9.102	11.114	3.163	Increase
18	Coniferyl acetate	223.0956	5.44	M+H	Pos	C_12_H_14_O_4_	222.24	0.0000	0.818	0.748	0.520	Decrease
19	Coniferyl alcohol	181.0513	3.10	M+H	Pos	C_10_H_12_O_3_	180.20	0.0060	3.781	4.888	1.280	Increase
20	Coniferyl aldehyde	179.0694	5.71	M+H	Pos	C_10_H_10_O_3_	178.18	0.0000	2.105	3.467	0.579	Increase
21	Coumarin	145.0289	4.56	M-H/M+H	Neg	C_9_H_6_O_2_	146.14	0.0000	1.995	1.259	1.569	Increase
22	Coumaroyl-glucose	327.1072	7.22	M+H	Pos	C_15_H_18_O_8_	326.30	0.0000	1.306	1.055	0.529	Increase
23	Coumaryl acetate	237.0776	4.15	M-FA	Neg	C_11_H_12_O_3_	192.21	0.0000	2.331	1.643	1.049	Increase
24	Cyanidin 3-(p-coumaroyl)-glucoside	610.1549	5.90	M-NH3	Neg	C_30_H_26_O_13_	595.53	0.0381	2.996	1.050	1.019	Increase
25	Dhurrin	334.0893	4.20	M+H_Na	Pos	C_14_H_17_NO_7_	311.29	0.0049	37.936	31.379	27.650	Increase
26	Dihydroconiferyl alcohol glucoside	411.1290	6.06	M-FA-Na	Neg	C_16_H_24_O_8_	344.36	0.8474	1.203	0.524	0.241	Increase
27	Dihydroxy-4-methoxy-isoflavanol	333.0991	3.91	M-FA	Neg	C_16_H_16_O_5_	288.30	0.0000	12.159	3.158	1.509	Increase
28	Dihydroxycinnamate	179.0341	5.29	M-H	Neg	C_9_H_8_O_4_	179.15	0.0000	1.466	0.950	0.495	Increase
29	Feruloyl-glucose	401.1081	3.19	M + FA	Neg	C_16_H_20_O_9_	356.33	0.0001	1.852	4.470	1.097	Increase
30	Fumarate	182.9915	1.75	M-FA-Na	Neg	C_4_H_4_O_4_	116.07	0.0000	0.742	1.037	0.907	Decrease
31	Geranyl-farnesyl diphosphate	591.1825	4.17	M-KCl	Neg	C_25_H_44_O_7_P_2_	518.57	0.0244	1.218	1.132	0.914	Increase
32	Gibberellin A9 methyl ester	375.1556	5.77	M+H_NaNa	Pos	C_20_H_26_O_4_	330.42	0.0000	2.565	1.847	1.221	Increase
33	Glutathione disulphide	611.1435	1.87	M-H	Neg	C_20_H_32_N_6_O_12_S_2_	612.63	0.0000	3.834	1.069	1.025	Increase
34	Hesperidin	609.1809	5.53	M-H	Neg	C_28_H_34_O_15_	610.57	0.0000	7.596	3.658	1.266	Increase
35	Homofuraneol	165.0523	1.83	M+H_Na	Pos	C_7_H_10_O_3_	142.15	0.0085	2.216	1.722	0.447	Increase
36	Hydroxybrassinolide	517.3128	8.17	M-Na	Neg	C_28_H_48_O_7_	496.69	0.0013	4.996	3.282	1.822	Increase
37	Hydroxyjasmonate	228.1593	6.98	M+H	Pos	C_12_H_18_O_4_	226.272	0.0100	1.422	1.519	0.309	Increase
38	Indole-3-acetaldoxime	175.0860	1.97	M+H	Pos	C_10_H_10_N_2_O	174.08	0.0009	1.140	1.719	0.809	Increase
39	Indole	116.0500	4.10	M-H	Neg	C_8_H_7_N	117.15	0.0079	3.591	1.373	2.933	Increase
40	Indole-3-acetamide	289.1181	4.88	M-H	Neg	C_15_H_18_N_2_O_4_	290.13	0.0123	1.222	2.001	1.006	Increase
41	Indole-3-acetyl-alanine	283.0479	4.78	M-K	Neg	C_13_H_14_N_2_O_3_	245.26	0.0000	0.793	0.906	0.692	Decrease
42	Indole-3-acetyl-beta-1-D-glucoside	337.0901	3.86	M	Pos	C_16_H_19_NO_7_	337.12	0.0000	8.370	6.306	3.136	Increase
43	Indole-3-glycerol phosphate	286.2377	11.07	M-H	Neg	C_11_H_14_NO_6_P	287.21	0.0000	0.661	0.674	0.439	Decrease
44	Indole-3-yl-acetyl-myo-inositol-L-arabinoside	468.1489	4.52	M-H	Neg	C_21_H_27_NO_11_	469.44	0.0023	1.662	1.552	0.938	Increase
45	Isoliquiritigenin 4'-glucoside	463.1243	5.36	M-FA	Neg	C_21_H_22_O_9_	418.13	0.0000	2.316	2.625	1.062	Increase
46	Isovitexin 7-O-glucoside	593.1499	6.09	M-H	Neg	C_27_H_30_O_15_	594.52	0.0000	0.641	0.782	0.242	Decrease
47	Kaempferol 3,7-O-diglucoside	609.1450	5.99	M-H	Neg	C_27_H_30_O_16_	610.52	0.0089	5.498	3.325	1.349	Increase
48	Kaempferol 3-O-glucoside	449.1075	6.71	M-HH	Neg	C_21_H_19_${\text{O}}_{11}^{-}$	447.37	0.0000	5.777	1.550	1.042	Increase
49	Luteolin	287.0566	6.31	M+H	Pos	C_15_H_10_O_6_	286.24	0.0086	17.712	9.687	2.399	Increase
50	Luteolin 7-O-glucoside	447.0917	6.19	M-H	Neg	C_21_H_20_O_11_	448.37	0.0000	20.535	15.686	5.252	Increase
51	Luteolinidin	271.0620	6.87	M	Pos	C_15_H_11_${\text{O}}_{5}^{+}$	271.24	0.0001	31.158	20.078	12.412	Increase
52	Naringin	625.1770	4.49	M-FA	Neg	C_27_H_32_O_14_	580.54	0.0000	1.992	1.992	1.226	Increase
53	N-Feruloylserotonin	351.1335	11.68	M-H	Neg	C_20_H_20_N_2_O_4_	352.39	0.0005	0.701	0.802	0.402	Decrease
54	p-Coumaroylagmatine	275.1997	13.69	M-H	Neg	C_14_H_20_N_4_O_2_	276.34	0.0001	1.751	1.750	1.907	Increase
55	p-Coumaroylquinate	427.0619	1.81	M-FA-NaNa	Neg	C_16_H_18_O_8_	338.31	0.0013	0.940	0.460	1.599	Decrease
56	Pentahydroxychalcone 4'-O-glucoside	449.1067	5.39	M-H	Neg	C_21_H_22_O_11_	450.12	0.0000	2.597	1.545	0.545	Increase
57	Phenyl methanol	177.0528	4.97	M+H_FANa	Pos	C_7_H_8_O	108.14	0.0000	0.873	1.042	0.582	Decrease
58	Phenylalanine	164.0922	3.94	M-H	Neg	C_9_H_11_NO_2_	165.19	0.0172	0.210	1.614	0.206	Decrease
59	Phenylethylamine	142.0635	4.08	M-Na	Neg	C_8_H_11_N	121.18	0.0106	1.667	1.057	1.802	Increase
60	Quercetin 3-O-rhamnoside	447.0914	5.04	M-H	Neg	C_21_H_20_O_11_	448.38	0.0000	11.038	10.655	3.675	Increase
61	Quercetin 3-sulfate	426.9968	3.35	M-FA	Neg	C_15_H_10_O_10_S	382.30	0.0000	2.001	1.993	1.125	Increase
62	Quercetin-3-rhamnoside-7-rhamnoside	595.1655	5.24	M-H	Neg	C_27_H_32_O_15_	596.17	0.0499	9.950	7.112	1.013	Increase
63	Riboflavin	443.1183	5.58	M-FA-NaNa	Neg	C_17_H_20_N_4_O_6_	376.37	0.0081	1.864	0.941	0.641	Increase
64	Salicyl alcohol	147.0415	1.93	M+H_Na	Pos	C_7_H_8_O_2_	124.14	0.4953	2.743	1.517	0.945	Increase
65	Salicylate 2-O-beta-D-glucoside	137.0241	7.29	M-H	Neg	C_7_H_6_O_3_	138.12	0.0589	29.384	5.402	1.131	Increase
66	Sinapaldehyde glucoside	415.1247	5.68	M-FA	Neg	C_17_H_22_O_9_	370.35	0.0000	0.679	1.097	0.903	Decrease
67	Sinapoyl aldehyde	369.1187	6.02	M-H	Neg	C_17_H_22_O_9_	370.35	0.0687	5.986	2.775	1.837	Increase
68	Sinapoyl malate	385.0762	4.74	M-FA	Neg	C_15_H_16_O_9_	338.27	0.0138	0.711	1.140	1.000	Decrease
69	Sinapyl-alcohol	299.0520	7.60	M-FA-NaNa	Neg	C_11_H_14_O_4_	210.23	0.0000	1.499	1.694	1.006	Increase
70	Sophoraflavanone G	423.1834	5.39	M-H	Neg	C_25_H_28_O_6_	424.49	0.0000	1.613	0.984	0.118	Increase
71	Syringin	409.0908	4.37	M-K	Neg	C_17_H_24_O_9_	372.37	0.3814	0.897	6.358	0.636	Decrease
72	Tryptophan	205.0968	3.99	M+H	Pos	C_11_H_12_N_2_O_2_	204.23	0.0002	5.459	2.043	1.410	Increase

*These discriminating metabolites were identified based on OPLS-DA S-plots, all with p-values < 0.05 and VIP scores > 1.0. FC refers to fold change (5 d.p.i. vs. control); and B, S, and M refer to the BTT, SWT, and MHL cultivars, respectively*.

Biochemical networks were generated using the Cytoscape (www.cytoscape.org) version 3.5.0 tool. Structural similarities were determined based on similarities between PubChem (https://pubchem.ncbi.nlm.nih.gov/) Substructure Fingerprints (ftp://ftp.ncbi.nlm.nih.gov/pubchem/specifications/pubchem_fingerprints.txt). Molecular fingerprints, defined by the presence or absence of physical properties (e.g., element type, functional group, nearest neighbors) and PubChem compound identifiers (CIDs), were used to calculate structural similarities (Bolton et al., 2011). Molecular fingerprints were compared and a threshold for structural similarity was defined at a Tanimoto coefficient of 0.7 (Grapov et al., 2015). The developed network was visualized using Cytoscape (Shannon et al., 2003; Smoot et al., 2011), and network characteristic mapping was used to encode chemometric modeling information through the network edge and nodes attributes.

### Gene Expression Analyses

Total RNA was extracted from harvested leaf tissues, corresponding to the different time-points (1–9 d.p.i.) of each biological repeat, using the Trizol-reagent method (Invitrogen, Carlsbad, CA, USA). The extracted RNA samples were subjected to DNase treatment using DNase I (Thermo Scientific, Waltham, MA, USA). Concentrations were determined using a NanoDrop® ND-1000™Spectrophotometer (NanoDrop Inc., Wilmington, DE, USA). The RNA integrity of all samples were examined by electrophoresis on a 1.5% agarose gel in 1X Tris-Borate-EDTA (TBE) buffer and containing 0.5 μg mL^−1^ ethidium bromide before use. The gels were visualized under UV light using a Bio-Rad Image Analyser and Quantity One™ Version 4.6.1 Software (Bio-Rad Laboratories, Johannesburg, South Africa). The total RNA samples were aliquoted and stored at −80°C for later use.

Real time PCR (qPCR) was used for sorghum gene expression analysis. Prior to quantification of the expression levels, the DNase-treated RNA were reverse transcribed to cDNA using a RevertAid™ Premium First Strand cDNA synthesis kit (Fermentas, Thermo Scientific, Waltham, MA, USA). The selected genes included: *chitinase (PR3), pathogenesis-related protein 10* (*PR10*), *flavonoid 3*′*-hydroxylase* (*F3'H*), *phenylalanine ammonia-lyase* (*PAL*) and *polyphenol oxidase* (*PPO*). The gene-specific primer pairs (Supplementary Table S1) were designed using the “Primer Quest” tool (Integrated DNA Technologies, Coralville, IA, USA) from sequences obtained in on-line data bases (GenBank NCBI, www.ncbi.nlm.nih.gov/genbank). qPCR was performed to analyse the expression of each gene using a RotorGene-3000A instrument (Qiagen, Venlo, Netherlands) using the FastStart essential DNA Green Master Kit (Roche, Mannheim, Germany) according to the manufacturer's instructions. Ten micro liter of SYBR (FastStart essential DNA Green Master), 1 μL forward primer (1 μM final concentration), 1 μL reverse primer (1 μM final concentration), and 6 μL of DNase-free water were added to 2 μL of cDNA for amplification in a total volume of 20 μL. The cycling conditions were as follows: initial denaturation for 10 min at 95°C followed by amplification and quantification cycle repeated 40 times each consisting of 5 s denaturing at 95°C, 10 s annealing at primer specific temperatures, 20 s extension at 72°C. Two independent cDNA preparations were used with three technical replicates of each. Quantification of the relative changes in gene expression was performed using the relative standard curve method (Liu and Saint, 2002) with *elongation factor 1-alpha (Elf* α*)* and *ubiquitin conjugating enzyme 18* (UBC18) as references genes. Data sets were statistically compared with the statistical analysis software GraphPad InStat v3 (GraphPad software, San Diego, CA, USA) using one-way analysis of variation (ANOVA) with Dunnet's post-test comparison of all treated samples vs. non-treated samples (control) at each time point. The confidence level of all analyses was set at 95%, and values with *p* < 0.05 were considered significant.

## Results

### Evaluation of Anthracnose Symptoms Development—Symptomatology

The symptomatic observations regarding the development of symptoms and disease severity of the MHL, BTT and SWT cultivars (Supplementary Figures S3, S4 and Supplementary Table S2) point to cultivar-related differential interactions between the sorghum plants and the hemibiotrophic *C. sublineolum* pathogen.

### Metabolic Profiling of *C. sublineolum*-Induced Changes in Sorghum

**Figure 1:**
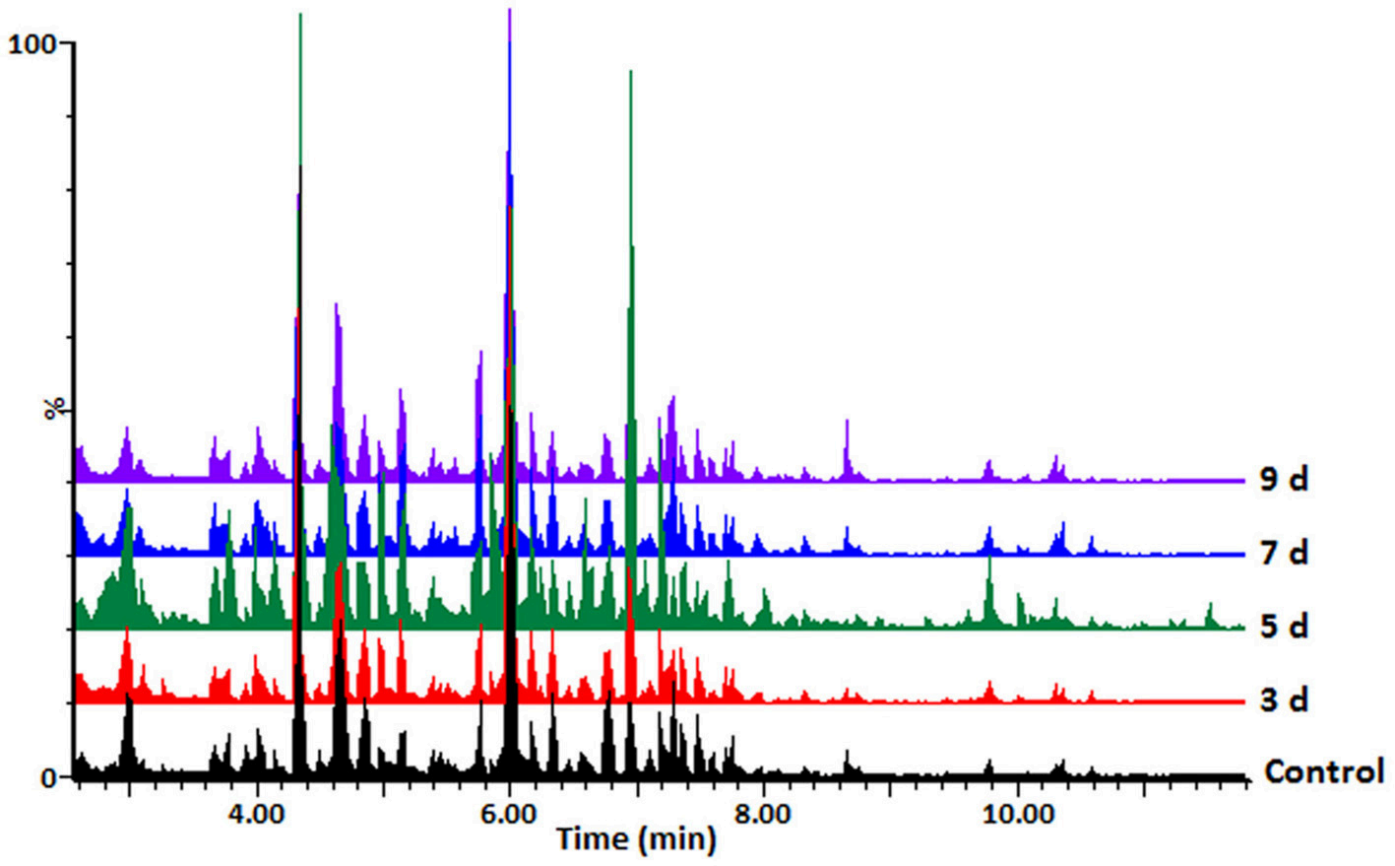
UHPLC-MS BPI chromatograms (ESI negative data): typical mass chromatograms of extracts from the sorghum NS 5511 (BTT) cultivar leaf tissue samples, responding to fungal infection. Control refers to samples from non-infected plants. The 3–9 d.p.i. samples are also indicated. Visual inspection of these mass chromatograms shows resolution of a number of ion peaks, reflecting the complexity of the extracts. Differential peak populations point to treatment-related metabolic changes: for instance in the retention time range of 4–8min, where differential peak intensities can be seen, and presence or absence of peaks.

Hydromethanolic extracts of *C. sublineolum*-infected and non-infected sorghum plants were analyzed on a reversed phase liquid chromatography (LC) column coupled to a high-resolution quadrupole time-of-flight (QTOF) mass spectrometry (MS) detector system with electrospray ionization (ESI). This LC-ESI-QTOF-MS platform was combined with an untargeted approach to gather information on as many statistically significant metabolites as possible. Considering the inherent chemo-diversity, heterogeneity, and multi-dimensionality of extracted metabolomes, chromatographic separation is an essential step in untargeted metabolomics workflow, providing resolution of sample constituents (Tugizimana et al., 2013). Interfaced in-line with ESI-MS, the resultant LC-MS analytical platform allowed the simultaneous detection of multiple analytes with high sensitivity, providing deeper and more detailed insight into the metabolic composition of a biological sample. Distinct MS chromatograms indicated differential metabolic profiles of the analyzed samples. Figure 1 and Supplementary Figures S5A,B shows typical base peak intensity (BPI) mass chromatograms with differential peak population (presence and intensities), reflecting differences between samples from infected and non-infected plants, as well as cultivar-related differences.

**Figure 2:**
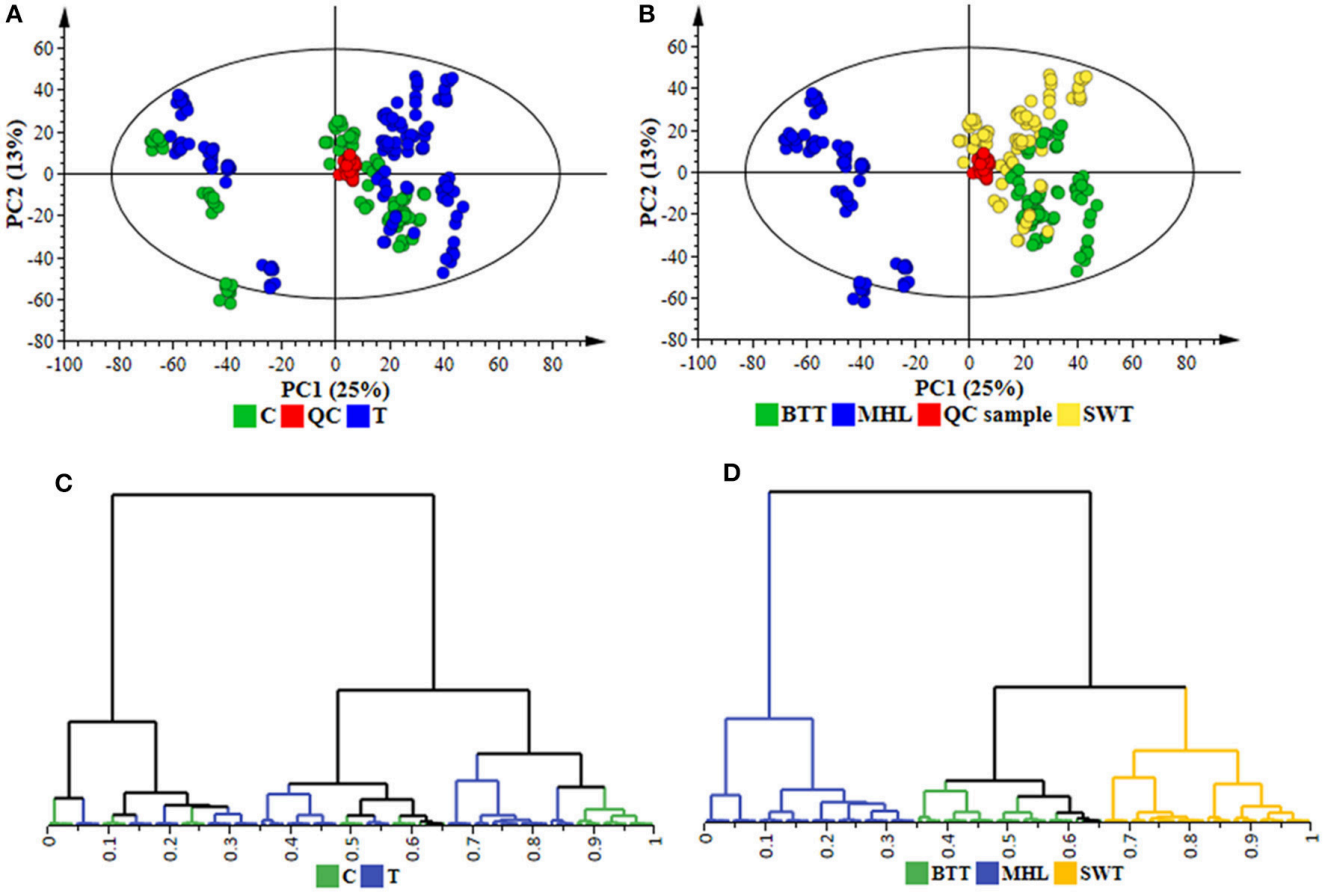
Unsupervised chemometric modeling (ESI negative data): **(A)** A PCA scores scatter plot of all the samples, including the QC samples, colored according to the treatment (C = control, T = treated). **(B)** The scores plot in **(A)** but colored according to cultivars (MHL = Mhlophe, BTT = NS 5511, SWT = NS 5655). The PCA model presented here was an 18-component model (of the Pareto-scaled data matrix **X**), with *R*^2^ of 0.739, explained variation, and Q^2^ of 0.664, predicted variation, according to seven-fold cross validation. **(C,D)** are HCA dendrograms corresponding to **(A,B)**, respectively. The unsupervised modeling provided a global overview of the data shown in the PCA scores plots and HCA dendrograms, allowing the identification of sample grouping and natural clustering with regards to treatment-related and cultivar-dependent groupings **(A**–**D)**.

To further elucidate the functional readouts of cellular physiological state(s) related to sorghum responses to *C. sublineolum* infection, chemometric analyses were applied to the collected LC-MS data. Following data processing (Boccard and Rudaz, 2014; Tugizimana et al., 2016), the created data matrices, with the number of defined features (Rt, *m/z*) being 1536 in ESI positive and 2759 in ESI negative data sets, were then exported into SIMCA (version 14) software for multivariate data analyses that included PCA, HCA, and OPLS-DA modeling. For the descriptive exploration of the overall structure of the pre-processed multi-dimensional data, unsupervised learning methods—PCA, and HCA—were used. These multivariate methods attempt to highlight descriptively trends and groupings within a data set, subsequently facilitating the understanding of the relationships between- and within the samples (Trygg et al., 2007; Tugizimana et al., 2013). PCA modeling, through the first two principal components (PCs), revealed treatment-related and cultivar-related sample clustering (Figures 2A,B and Supplementary Figure S6). Furthermore, in the PCA space, the QC samples are clustered closely to each other (and more or less in the middle of the 2D-plots), reflecting the stability of the LC-MS system used, and the reliability and reproducibility of the analysis (Godzien et al., 2015; Broadhurst et al., 2018). These sample groupings highlighted by the computed PCA models point to differential metabolic changes in sorghum plants responding to *C. sublineolum*. The samples from the MHL cultivar formed a clearly different group from the other two cultivars. This observation correlates to the indications from symptomatology (Supplementary Figures S3, S4 and Supplementary Table S2).

The PCA-extracted trends in the data were further examined by applying hierarchical clustering analyses on low-dimensional data generated from the PC analyses. Agglomerative HCA models were computed using Ward's linkage method (incremental sum of squares method) that considers between- and within-cluster distances when forming clusters, and the tree was sorted based on size (Szekely and Rizzo, 2005; Ji and Liu, 2010). The generated hierarchy of clusters was represented graphically on a dendrogram to evaluate whether some natural grouping emerges from the data—i.e., if the “metabolite space” actually contains several distinct subspaces. The computed HC models depicted two major distinct clusters corresponding to the samples from the very susceptible MHL cultivar grouping differentially and separate from the other two cultivars (BTT and SWT). Treatment-related (infected vs. non-infected) and time-related sub-clusters were also formed within each major cluster (Figures 2C,D and Supplementary Figure S6). Thus, both PCA and HCA modeling aided to evaluate descriptively the overall structure of the data, revealing underlying patterns and inner structures and sub-structures within the data: cultivar-related clustering, treatment-dependent groupings (infected vs. non-infected), and time-related variation (Figures 2C,D and Supplementary Figure S6). These observations evidently point to a biological phenomenon in the (extracted) metabolite space—differential metabolite profiles defining temporal cellular events related to the sorghum plants' responses to *C. sublineolum* infection.

**Figure 3:**
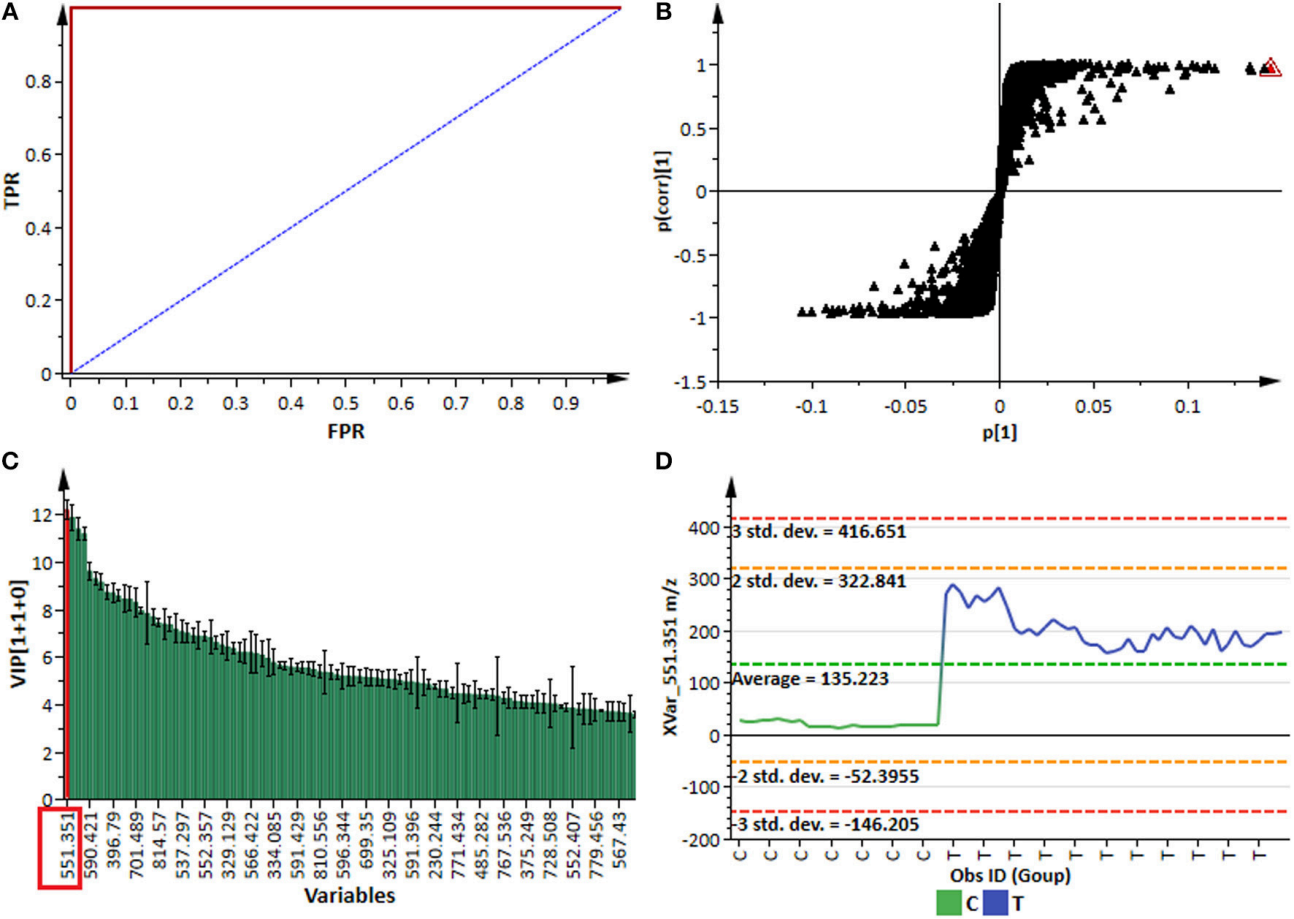
OPLS-DA modeling and variable/feature selection (data from NS 5511/ BTT cultivar samples). **(A)** A typical receiver operator characteristic (ROC) plot for the OPLS-DA model (ESI negative data) separating “control vs. infected plants” at 7 d.p.i. (1 + 1 + 0 components, *R*^2^X = 0.611, Q^2^ = 0.994, CV-ANOVA *p*-value = 2.4 × 10^−14^). The ROC plot is a graphical summary of the performance of a binary classifier. A model with perfect discrimination has a ROC curve with 100% sensitivity and 100% specificity, as it is the case with this OPLS-DA model. **(B)** An OPLS-DA loadings S-plot for the same model in **(A)**; variables situated in the extreme end of the S-plot are statistically relevant and represent prime candidates as discriminating variables/features. **(C)** A variable importance for the projection (VIP) plot for the same model; pointing mathematically to the importance of each variable (feature) in contributing to group separation in the OPLS-DA model. **(D)** A typical variable trend plot (of the selected variable in VIP and S-plots), displaying the changes of the selected variable across the samples. C = control; and T = treated samples (7 d.p.i.). The variable trend plot show that the selected feature significantly discriminates the treated from the control samples.

For better biochemical interpretability and detailed assessment of the metabolic changes revealed by PCA and HCA in sorghum responding to the fungal infection, the supervised modeling method, OPLS-DA, was used. Evaluation of this multivariate (binary) classifier helps in extracting the metabolite variables underlying the discrimination between classes or groups (Trygg et al., 2007; Tugizimana et al., 2013). OPLS-DA is an extension to the supervised PLS-DA regression method, featuring an integrated orthogonal signal correction (OSC)-filtering method and, as such, OPLS-DA modeling has added interpretational and discriminatory benefits compared to PLS-DA (Bylesjö et al., 2006). The computed OPLS-DA models (Figure 3 and Supplementary Figure S7) to separate multivariate relationships into predictive (related to *C. sublineolum* infection) and orthogonal (unrelated to the treatment) variation, were validated with multivariate statistical tools and scrutinized by assessing the robustness, predictive ability, reliability and significance of the models. Some of the multivariate statistical tool used to validate calculated OPLS-DA models included *R*^2^–and Q^2^ metrics, the analysis of variance testing of cross-validated predictive residuals (CV-ANOVA, *p*-value <0.05 as a cut-off), the receiver operator characteristic (ROC) curves, response permutation tests (with *n* = 50), and predictive testing (Eriksson et al., 2008; Tugizimana et al., 2016). The computed and validated OPLS-DA models (*p* < 0.05) used in this study were perfect classifiers and statistically reliable, with very good predictive capability: no signs of possible overfitting, as indicated by cross-validation; and none of the permutated models performed better than the original models in separating classes (Figure 3 and Supplementary Figure S7). These binary classifier models allowed to assess explicatively the treatment-related groupings (described by the unsupervised PCA and HCA models above) by extracting features (variables) responsible for differentiating sample groups (e.g., infected vs. non-infected).

Thus, the selection of discriminating features (signatory biomarkers characterized by unique Rt and *m/z* values) was carried out by evaluating the OPLS-DA loading S-plots (Figure 3B). To avoid overinterpretation of the models and variable selection bias, only features that were statistically significant in contributing to class separation were retained. Therefore, variables that combined both high covariation and correlation (as examined on S-plots) were considered to be statistically relevant as potential discriminant features (Wiklund et al., 2008; Tugizimana et al., 2013). However, since the S-plot is susceptible to data matrix changes due to correlation sensitivity and dependency on data structure, the statistical significance and discriminability of the potential markers derived from the S-plots were further investigated using different tests and tools such as the VIP plots, jackknife confidence intervals (used to estimate standard errors in a non-parametric way as an estimate of bias), variable trends, dot plots and descriptive statistics (Figures 3C,D). The VIP plots display VIP values as a column plot with jackknife uncertainty bars, providing a metric to assess the importance of the variables both to explain **X** and to correlate to **Y**, with the jackknife confidence intervals reflecting the variable stability (Galindo-Prieto et al., 2015; Tugizimana et al., 2016). Only S-plot-derived variables with VIP scores exceeding 1.0, with no (or minimal) overlap between groups (as indicated by dot—and trends plots), with positive jackknife confidence intervals and *p*-value < 0.05 (ANOVA, *T*-test), and demonstrating stable signals in the QC samples, were selected and retained as statistically significant and chemometrically contributing correctly to class separation. In a logical extension, these selected discriminant variables are regarded as essential chemical repertoires explaining the metabolic changes in sorghum, revealed by PCA—and HCA models (Figures 2C,D and Supplementary Figure S6). Accordingly, such features are fundamental elements for the biochemical interpretation of the chemometrically extracted information. These extracted features (markers) were then annotated (to the Metabolomics Standards Initiative, MI-level 2 annotation), as described in the experimental section and are reported in Table 1.

### Metabolite Pathway Analysis and Metabolic Network Analysis

**Figure 4:**
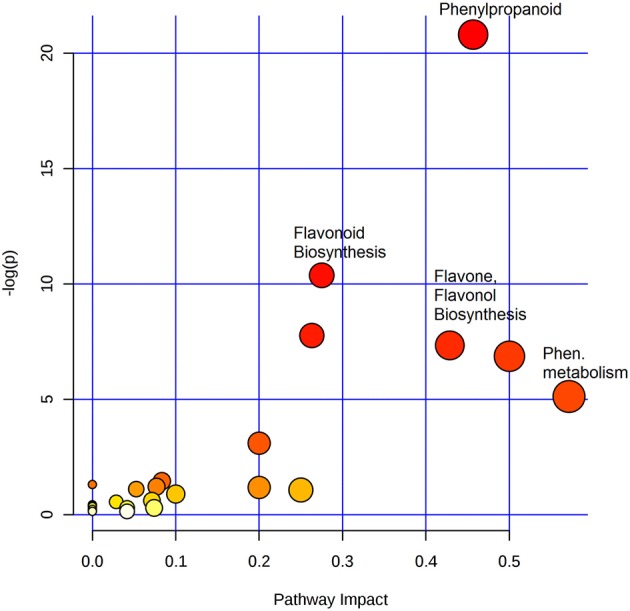
Summary of pathway analysis with MetPA: Representation of all MetPA-computed metabolic pathways displayed according to their significance or pathway impact. The graph, presents a view of all the matched pathways arranged by *p*-values (pathway enrichment analysis) on the *y*-axis, and the pathway impact values (pathway topology analysis) on the *x*-axis. The node color (beige to red) is based on the node's *p*-value and the node radius is defined by the pathway impact values. The latter is the cumulative percentage from the matched metabolite nodes, and the maximum importance of each pathway is 1. Thus, the graph indicates pathways with high impact: phenylalanine metabolism, phenylpropanoid-, flavonoid-, flavone- and flavonol-biosynthesis, to be highly significant metabolic pathways that are involved in the sorghum response to *C. sublineolum* infection.

**Figure 5:**
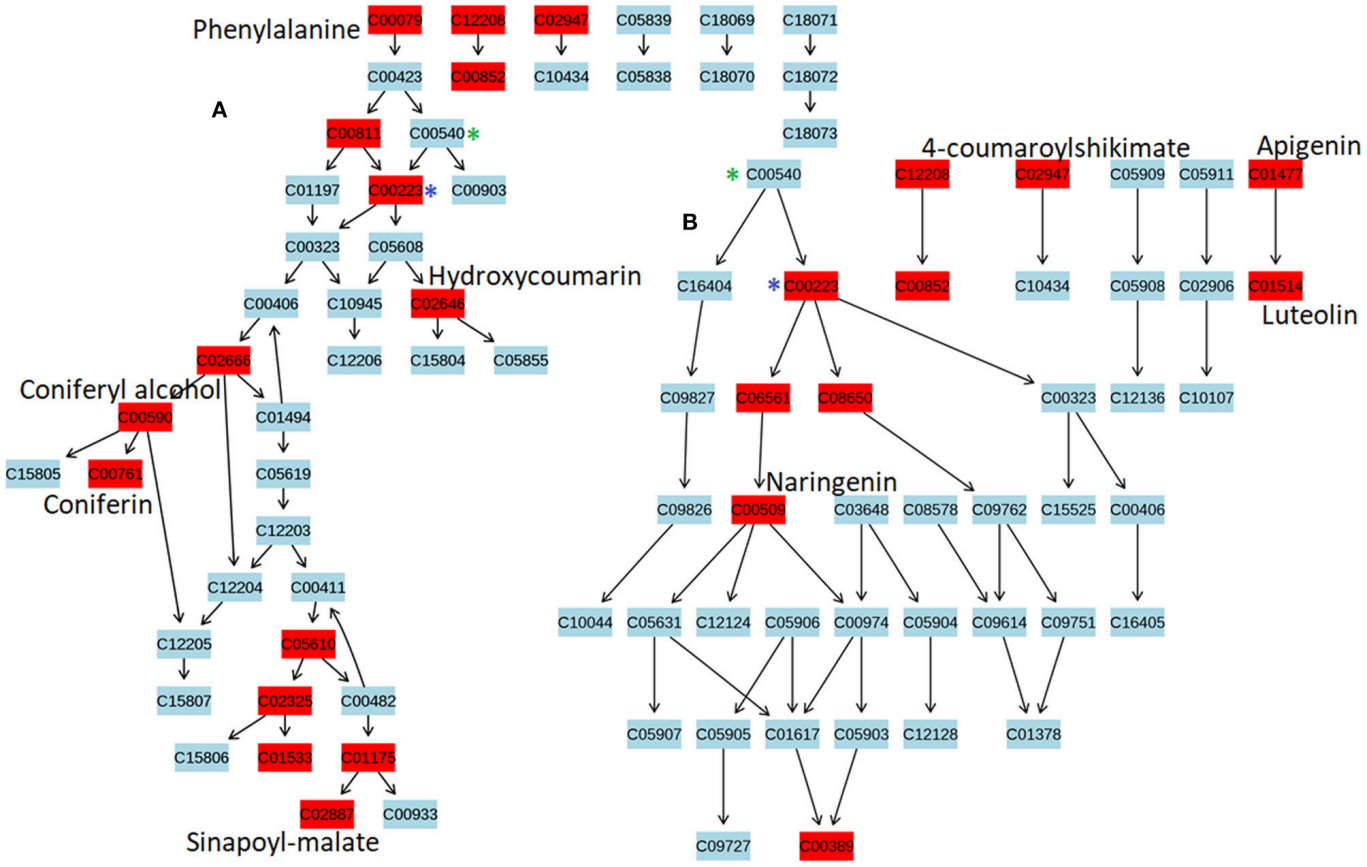
The topological characteristics of phenylpropanoid and flavonoid pathways. Graphs were generated from Metabolomics Pathway Analysis using MetPA. **(A)** *Phen*ylpropanoid pathway map displaying some of the OPLS-DA selected metabolites, altered in response to *C. sublineolum* infection. **(B)** Flavonoid pathway map showing OPLS-DA selected metabolites altered during sorghum responses to *C. sublineolum* infection. (^*^) indicate overlapping points where metabolic pathways interconnect. Due to the limitation of the MetPA tool (considering the database used and search algorithm) not all metabolites that were chemometrically extracted (Table 2) could be mapped in the constructed pathway graphs.

To identify the most significant metabolic pathways defining the sorghum defense responses, the MetPA (Metabolomics Pathway Analysis)—an integral module of the MetaboAnalyst bioinformatics tool suite (version 3.0; http://www.metaboanalyst.ca/)—was used. MetPA is a pathway analysis and visualization tool that combines several advanced pathway enrichment analysis methods along with the analysis of pathway topological characteristics to facilitate the elucidation of most relevant and altered pathways involved in the conditions under study (Xia et al., 2015; Chong et al., 2018. A representation of all MetPA-computed metabolic pathways displayed according to their significance or pathway impact in shown in Figure 4 and Table 2. The nine most significant pathways were (with some overlap): phenylalanine metabolism, stilbenoid and gingerol biosynthesis, flavonoid biosynthesis, flavone and flavanol biosynthesis, tryptophan metabolism, phenylpropanoid biosynthesis, aromatic amino acid biosynthesis, riboflavin-, and tyrosine metabolism. Furthermore, the topological characteristics of the phenylpropanoid—and flavonoid pathways are shown in Figure 5, illustrating that the two pathways are structurally highly interconnected, with some overlap as also shown in the topological graph-pathways generated from MetPA.

**Table 2:** Significant metabolic pathways activated in sorghum responding to *C. sublineolum* infection, inferred from Metabolomics Pathway Analysis (MetPA).

**No**	**Pathway Name**	**Total**	**Expected**	**Hits**	**Impact**
1	Phenylalanine metabolism	8	0.41	3	0.67
2	Stilbenoid, diarylheptanoid, and gingerol biosynthesis	10	0.51	4	0.50
3	Flavonoid biosynthesis	43	2.20	10	0.46
4	Flavone and flavonol biosynthesis	9	0.46	4	0.44
5	Tryptophan metabolism	27	1.38	4	0.34
6	Phenylpropanoid biosynthesis	45	2.31	15	0.30
7	Phenylalanine, tyrosine, and tryptophan biosynthesis	21	1.08	6	0.24
8	Riboflavin metabolism	10	0.51	1	0.20
9	Tyrosine metabolism	18	0.92	2	0.18
10	Glutathione metabolism	26	1.33	1	0.05
11	Citrate cycle (TCA cycle)	20	1.02	1	0.03
12	Carotenoid biosynthesis	37	1.90	2	0.01
13	Alanine, aspartate, and glutamate metabolism	22	1.13	1	0.01
14	Diterpenoid biosynthesis	26	1.33	1	0.01
15	Isoquinoline alkaloid biosynthesis	6	0.31	1	0.00
16	Indole alkaloid biosynthesis	7	0.36	1	0.00
17	Ubiquinone and other terpenoid-quinone biosynthesis	23	1.18	2	0.00
18	Tropane, piperidine, and pyridine alkaloid biosynthesis	8	0.41	1	0.00
19	Nitrogen metabolism	15	0.77	1	0.00
20	Alpha-Linolenic acid metabolism	23	1.18	1	0.00
21	Glycine, serine, and threonine metabolism	30	1.54	1	0.00
22	Arginine and proline metabolism	38	1.95	1	0.00
23	Aminoacyl-tRNA biosynthesis	67	3.43	2	0.00

**Figure 6:**
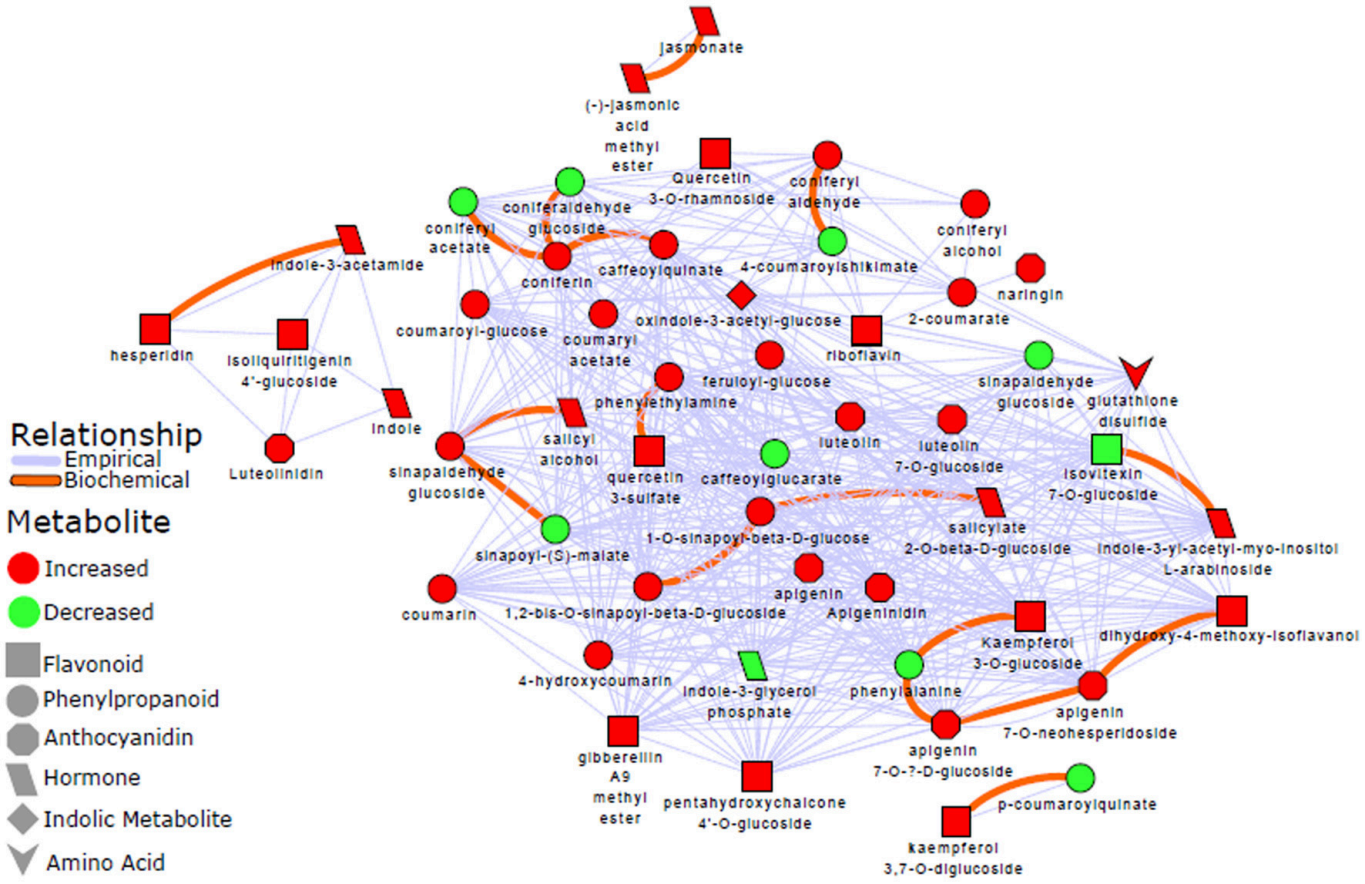
Metabolic network analysis: A biochemical and empirical network displaying metabolic relationship patterns between metabolites indicated by OPLS-DA as signatory biomarkers. Metabolites are connected based on biochemical relationships (red, KEGG RPAIRS) or structural similarity (blue); as also indicated on the network descriptive legend (left-side). Metabolite color represents relative change (red: increase; green: decrease) in infected plants compared to non-infected sorghum plants. The different shapes indicate the metabolites' molecular classes or biochemical domains. The graph was visualized with Cytoscape version 3.5.0. Applying a Tanimoto coefficient ≥0.7 for structural similarity, the resultant graphic representation and computed network parameters (e.g., clustering coefficient of 0.695; network density of 0.573) revealed a high interconnectivity of the OPLS-DA selected metabolites.

To complement these results, a biochemical and empirical network displaying metabolic relationship patterns between metabolites (indicated by OPLS-DA as signatory biomarkers) were performed. Figure 6 illustrates how the metabolites are connected based on biochemical relationships or structural similarity. The graphic representation and computed network parameters (e.g., clustering coefficient of 0.695; network density of 0.573) revealed a high interconnectivity of the OPLS-DA selected metabolites.

### Expression Analyses of Selected Defense-Related Genes in *Sorghum bicolor*

**Figure 7:**
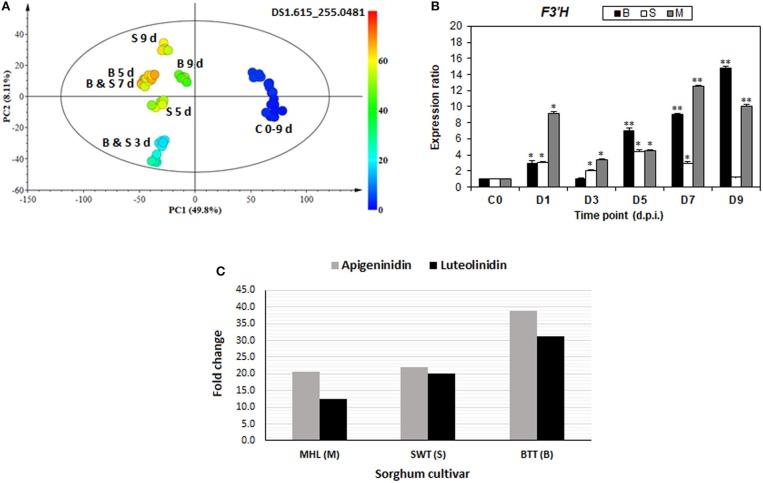
*De novo* biosynthesis of 3-deoxyanthocyanidin phytoalexins: **(A)** A color-coded PCA scores plot showing occurrence and increasing level of apigeninidin in infected sorghum plants. **(B)** Time-course analysis of relative expression of the *F3*′*H* gene (one of the key enzymes in the biosynthesis of 3-deoxyanthocyanidins) in infected sorghum plants (^*^ and ^**^ indicate significance at *p* < 0.05 and *p* < 0.001, respectively). **(C)** Relative fold changes of apigeninidin and luteolinidin, respectively, in the three sorghum cultivars at 7 d.p.i.

**Figure 8:**
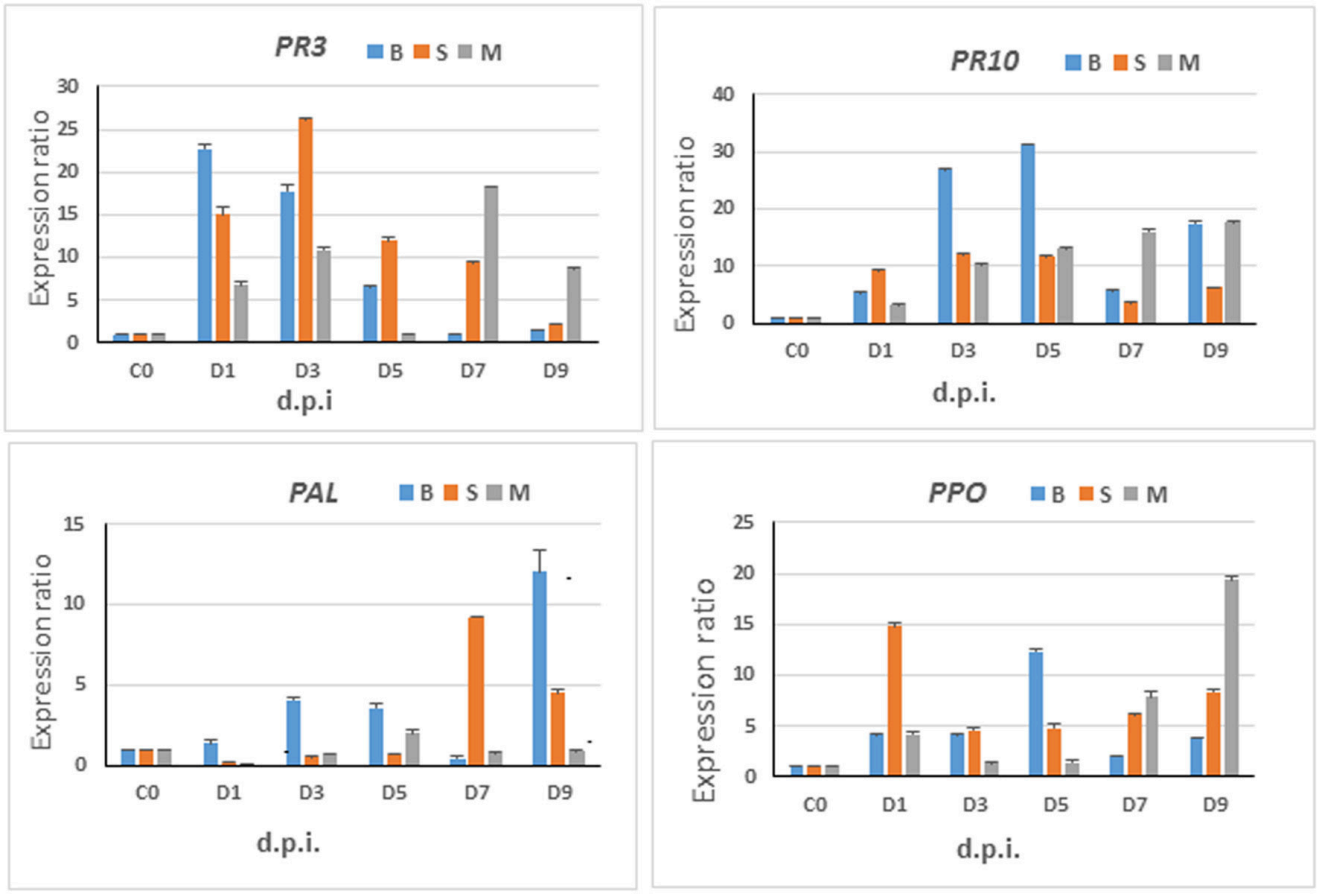
Gene expression analyses—relative expression of selected marker genes. Time-course studies of relative expression of *PAL, PPO, PR3*, and *PR10* genes in sorghum plants infected by *C. sublineolum*. M = Mhlophe/MHL, B = NS 5511/BTT, and S = NS 5655/SWT cultivars. Bars indicate the mean values and error bars indicate the standard deviation. Results were analyzed using ANOVA, with confidence level of 95% and significance level at *p* = 0.05.

To enrich the metabolomic results with transcriptome insights, a phytoalexin-related gene (*F3*′*H*) and some defense-related genes (*PAL* and *PPO*, and *PR-proteins PR3 and PR10*) were selected and analyzed for expression levels in response to *C. sublineolum* infection. The general observation from these results points to time- and cultivar-related expression profiles; and all genes showed significant expression levels at different time intervals after pathogen inoculation (Figures 7, 8).

## Discussion

### Evaluation of Anthracnose Symptoms Development—Symptomatology

The phenotypic observations can be interpreted as BTT exhibiting a stronger defense response than SWT, with hypersensitive response-like lesions and purple color formation around the infected tissue. In contrast, the MHL cultivar exhibited little or no resistance against *C. sublineolum*. As mentioned, anthracnose development and severity in sorghum vary depending on the interaction between *C. sublineolum* (variation in virulence within the pathogen population), the genetics-based potential of the host plant to ward off infection, as well as environmental conditions (Tesso et al., 2012). Considering that in this study the sorghum cultivars were infected with a genetically uniform *C. sublineolum* isolate, in a controlled environment, the symptomatic differences thus reflect cultivar-related responses to the fungal infection. Although the plant defense responses triggered upon fungal infection are broadly similar across cultivars, the kinetics of these biochemical and cellular events and the relative abundance and timing may vary among cultivars (Liu et al., 2010; Tesso et al., 2012). Furthermore, such observation points to the inherent complexity of multi-layered plant innate immunity, of which omics-based studies have barely scratched the surface. Hence, characterizing metabolic phenotypes related to the sorghum defense responses to *C. sublineolum* infection would provide more insights into cellular pathways linked to underlying biochemical and molecular mechanisms operative in this specific plant-pathogen interaction.

### Defense-Related Metabolic Reprogramming in *Sorghum bicolor*

For biochemical interpretation of the post-infection metabolic reprogramming in sorghum plants, as infographically described by the chemometric models, the statistically selected biomarkers/metabolites (Table 1) were further explained in the global metabolic interrelationships. Thus, metabolic pathway mapping and correlation network analyses were performed to elucidate the most relevant pathways and global dynamic metabolic networks involved in sorghum's responses to *C. sublineolum* infection. Both approaches exploited the relational properties present in the generated metabolomic data. Metabolic pathway analysis (or mapping) uses prior biological knowledge to map and analyse metabolites in an integrative manner, inferring significant pathways related to the study (Barupal et al., 2018; Rosato et al., 2018). On the other hand, metabolic network analysis methodology uses the high degree of correlation (biochemical and/or structural) existing in the generated metabolomic data to construct networks that characterize the complex relationship in measured metabolites (Toubiana et al., 2013; Grapov et al., 2015; Rosato et al., 2018).

Based on the chemometrically extracted metabolites (Table 1), pathway analysis with MetPA revealed that nine significant metabolic pathways out of a total of 24 pathways (impact score >0.10) were uniquely altered during the sorghum: *C. sublineolum* interactions. These most significant pathways include phenylalanine metabolism, flavonoid biosynthesis, phenylpropanoid biosynthesis, tryptophan metabolism, and riboflavin metabolism, among others (Figure 4 and Table 2). These results suggest that sorghum responses to *C. sublineolum* involves highly complex cellular reprogramming characterized by altered metabolism spanning a number of metabolic pathways; particularly the 9 significant pathways indicated in Table 2 and Figure 4, with phenylpropanoid and flavonoid biosynthesis pathways showing the highest hits. The constituents of these two metabolic pathways are the most widely occurring secondary metabolites found in the plant kingdom, exhibiting a broad range of biological functions including development, protection against abiotic and biotic stresses, modulation of essential physiological, and biochemical processes such as signal transduction, and transcriptional regulation (Cheynier et al., 2013; Petrussa et al., 2013).

Structurally, metabolites of phenylpropanoid and flavonoid pathways are phenolic compounds comprising an aromatic ring, with one or more hydroxyl groups, and include simple phenolic molecules to highly polymerised and conjugated compounds (Quideau et al., 2011; Petrussa et al., 2013). The two pathways are thus structurally highly interconnected, with some overlap as also shown in the topological graph-pathways generated from MetPA (Figure 5). Both pathways start with the conversion of phenylalanine to p-coumaroyl-CoA by phenylalanine ammonia-lyase (PAL), cinnamate-4-hydroxylase (C4H) and 4-coumaroyl:CoA-ligase (4CL) (Petrussa et al., 2013). The differentiation of the pathways rises with the formation of basic ring systems, with flavonoids showing a common three ring chemical structure (C_6_-C_3_-C_6_). The wide diversity of both phenylpropanoids and flavonoids is then brought about by efficient decoration, modification, and amplification of core structures by a set of enzymes that are spatially and temporally coordinated and highly regulated (Quideau et al., 2011; Petrussa et al., 2013). The widespread presence of the phenolic compounds (both from phenylpropanoid and flavonoid pathways) at cellular, tissue and organ level is a clear indication of the multiple biological—and biochemical functions in plants Quideau et al., 2011; Petrussa et al., 2013) and sorghum is found to contain an array of these phenolic compounds (Althwab et al., 2015; Kang et al., 2016). Studies have shown that these phenolics play crucial roles in plant-fungal interactions as protectants: either as pre-formed antifungal compounds (phytoanticipins) or induced antifungal molecules (phytoalexins; Lattanzio et al., 2006). As presented in Figure 5, the topological characteristics of phenylpropanoid and flavonoid pathways display some of the phenolic compounds (in red) that exhibited dynamic changes, also providing some insights into the relational properties.

Thus, differential metabolite changes (Table 1 and Supplementary Figures S8, 9) observed in this study, evidently indicate that following the perception of *C. sublineolum* invasion, sorghum launched a complex arsenal of chemical defenses. These involved changes in phytohormone levels, indole compounds and modulation and activation of the pre-existing antifungal (phenolic) metabolites, as well as *de novo* biosynthesis and translocation of (flavonoid) phytoalexins. The metabolic reprogramming was cultivar dependent and was typically exemplified by the identification of quantitative changes in jasmonic acid (JA)- and salicylic acid (SA) conjugates, abscisic acid (ABA) and in the constitutive metabolites such as naringin, quercetin, and its conjugates (e.g., quercetin 3-O-rhamnoside), kaempferol di-glucoside and coumarate and sinapoyl conjugates, among others (Figure 5, Table 1, Supplementary Figures S8, 9). The phytohormones coordinate multiple physiological and biochemical processed in plants, such as growth, development and responses to abiotic and biotic stresses. The intricate web of crosstalk between various plant hormones, either through synergistic or antagonistic interactions, fine-tunes the regulation of plant immune responses, and is linked to biotrophic- or necrotrophic pathogen lifestyles (Shigenaga and Argueso, 2016). The occurrence of and cultivar-related changes in JA, SA, and ABA in response to infection by hemibiotrophic *C. sublineolum*, point to a multicomponent sorghum defense response (Table 1, Supplementary Figure S8).

In previous studies of different phytopathosystems, some of the phenolic metabolites, found to differentially accumulated in this study, have been reported to be involved in defense mechanisms: naringin has been documented to show antifungal activity, acting as a defense barrier. Moreover, quercetin -, naringenin -, and kaempferol conjugates have been shown to exhibit biological activity against fungal pathogens and a significant inhibition of spore germination (Lattanzio et al., 2006; Cheynier et al., 2013). If the levels and types of pre-existing antifungal phenolics are not enough to effectively limit the infection process, plant cells would respond by altering the pool of these phenolics, by increasing the levels or structurally modifying these phenolics to biochemically activate the compounds. This alteration in phenolics metabolism provides adequate substrates to polyphenol oxidase-catalyzed reactions that produce an environment unfavorable to fungal pathogenicity (Dicko et al., 2005; Lattanzio et al., 2006; Constabel and Barbehenn, 2008). The responses of sorghum to *C. sublineolum* infection by altering the phenolic matrix (as shown in this study—Figure 5, Tables 1, 2, Supplementary Figure S9) thus demonstrates the onset of active defense mechanisms by sorghum to stop the fungal invasion. This correlates to previous studies that observed deposition of phenolics in sorghum leaves and stalks of both resistant and susceptible varieties post *C. sublineolum* infection (Dicko et al., 2005; Anjum et al., 2013). Furthermore, the quantitative assessment of these phenolics indicates cultivar-dependent responses: overall, the NS5511 (bitter, BTT) cultivar had higher levels of phenolics compared to other cultivars, whereas the Mhlope (sweet white, MHL) cultivar showed low levels of these defense-related metabolites (Supplementary Figure S9). This correlates to the symptomatology observations: the MHL cultivar appeared to be severely affected by the *C. sublineolum* infection (Supplementary Figure S4).

In addition to the other observed changes in the flavonoid pathways, the *de novo* biosynthesis of 3-deoxyanthocyanidin phytoalexins, apigeninidin, and luteolinidin, together with the related flavonoids such as apigenin, apigenin conjugates, luteolin and luteolin di-glucoside (Figures 5, 6; Table 1, Supplementary Figure S9) were observed. As infographically captured by the unsupervised color-coded-PCA scores plot (Figure 7A), for instance, the presence and abundance (expressed as integrated peak area in the data matrix X) of the apigeninidin molecular feature started appearing from 3 d.p.i. with a clear increase over time. This evidently showed that in non-infected sorghum plants (at 0–9 d) there was no detectable presence of apigeninidin, and only infected plants were seen to have synthesized this phytoalexin, with increasing levels over time. The accumulation of these antifungal phytoalexins—apigeninidin and luteolinidin—was further confirmed by gene expression analysis of *flavonoid 3*′*-hydroxylase* (*F3*′*H*), which showed a time-related increasing expression profile (Figure 7B). The *F3*′*H* gene encodes one of the key enzymes responsible for the biosynthesis of these 3-deoxyanthocyanidin phytoalexins, unique antifungal compounds synthesized by sorghum (and related plants such as sugar cane) after fungal infection. The F3'H enzyme is responsible for the multi-step biochemical formation of 3-hydroxyantocyanidins from naringenin (Boddu et al., 2004; Liu et al., 2010; Poloni and Schirawski, 2014). Flavonoid hydroxylases are microsomal cytochrome P450 enzymes responsible for hydroxylation patterns of flavonoids. The expression of *F3'H* gene is induced by fungal infection and responsible for the biosynthesis of 3-hydroxylated flavonoids, as shown in Supplementary Figures S1, S2 (Boddu et al., 2004; Petrussa et al., 2013). Thus, the expression of the *F3'H* gene in sorghum responding to *C. sublineolum* supports and confirms the metabolic results, that is the presence and accumulation of these 3-deoxyanthocyanidin phytoalexins. Previous studies have also indicated that sorghum responses to fungal infection are characterized by the accumulation of an array of phenolic compounds, with major components being the phytoalexins—apigeninidin and luteolinidin—and their conjugates, arabinosyl-5-O-apigeninidin, 7-methylapigeninidin, and 5-methoxyluterolinidin (Liu et al., 2010; Poloni and Schirawski, 2014). These experiment-based observations correlate with our results regarding the biosynthesis of phytoalexins apigeninidin, luteolinidin, and other related flavonoids (Figure 7, Table 1, Supplementary Figure S9). Functionally, these unique class of flavonoid phytoalexins are initially synthesized in the cytoplasm of epidermal sorghum cells following fungal infection, and accumulate in inclusion bodies. These are translocated toward the site of fungal invasion, where they are then released in active form and kill both the fungus and cells that synthesized them (Poloni and Schirawski, 2014; Meyer et al., 2016). The kinetics and intensity of this response appear to be cultivar-dependent, as reflected by the results in Figure 7C (and Supplementary Figure S9), showing comparative cultivar-related differences in the fold change of apigeninidin and luteolinidin, respectively.

The observations from this study and supported by the literature, point clearly to a complexly coordinated and highly regulated dynamic (and cultivar-dependent) metabolic reprogramming in sorghum responding to *C. sublineolum* infection. The functionally altered sorghum metabolism involved a range of different metabolic pathways (Table 2), which exhibit a complex interconnection as illustrated by the phenylpropanoid and flavonoid pathways (Figure 5). Pathway enrichment and overrepresentation analyses with the MetPA tool thus facilitated biochemical interpretation by integrating biological domain knowledge (i.e., biochemical pathways) with experimental results, revealing underlying relevant metabolic pathways (Figure 4 and Table 2). However, these pathway-based methodologies rely on predefined pathways, and fail for instance to capture linkage information of metabolites belonging to multiple pathways. This may not accurately represent the complexity of biological systems, subsequently providing limited insights into underlying mechanisms in the crowded cellular milieu, and spatial and temporal regulation of organismal reprogramming (Kruger and Ratcliffe, 2012; Toubiana et al., 2013; Barupal et al., 2018).

Hence, to gain more insights into possible global biochemical and molecular frameworks that choreograph the response of sorghum to *C. sublineolum* infection, a network analysis approach was adopted. This methodology uses the high degree of correlation (biochemical and empirical, in this study) existing in the experimentally generated metabolomic results to construct networks that characterize the complex relationship in measured metabolites (Kruger and Ratcliffe, 2012; Toubiana et al., 2013). Unlike pathway analysis, correlation-based approaches build metabolite networks according to relational patterns observed in the experimental data, and help identify altered graph neighborhoods, which do not depend on any predefined biochemical pathways. Such mathematically constructed cartography allows the characterization of the molecular and cellular states induced by pathway interconnections under given experimental conditions. In the computed network, each metabolite is represented by a node, and in contrast to pathway analysis, the links between nodes correspond to the level of mathematical correlation between each pair of metabolites (Kruger and Ratcliffe, 2012; Toubiana et al., 2013; Rosato et al., 2018). A biochemical/chemical similarity network analysis was accordingly applied to calculate and display relationship patterns between precursor and product metabolite reactant pairs, and molecules sharing a high degree of structural similarity, with Tanimoto coefficient ≥0.7. The resultant graphic representation and computed network parameters (e.g., clustering coefficient of 0.695; network density of 0.573) revealed a high interconnectivity of the OPLS-DA selected metabolites (Figure 6). These were statistically shown to explain class separation (e.g., infected vs. non-infected), thus describing the metabolic changes in sorghum revealed by the PCA and HCA models (Figures 2 and Supplementary Figure S6). Phenylpropanoids and flavonoids appear to form a central hub in the topology of the network, also maintaining high interactions with other metabolite categories, such as indolic or amino acid related metabolites (Figure 6).

Furthermore, the network analysis showed that separate from the major cluster, there were other small clusters that were formed: (i) luteolinidin, indole, indole-3-acetalamide, and hesperidin, (ii) kaempferol-diglucoside and *p*-coumaroylquinate, and (iii) jasmonate and jasmonic acid methyl ester (Figure 6). This differential clustering may possibly suggest different regulation of these metabolites in the concerted metabolic reprogramming of sorghum defense responses to the fungal infection. The computed high interconnectivity of nodes in the network demonstrates highly correlated biochemical and structural metabolic relationships that coordinate the altered sorghum metabolism in sorghum: *C. sublineolum* interactions. The significant metabolic pathways underlying sorghum responses to the fungal infection as revealed by pathway analysis with MetPA (Figures 4, 5 and Table 2), are highly interconnected, as demonstrated by the network topology (Figure 6). This points to regulatory hubs in the biochemical network (different clusters in the network), because the correlation matrix of metabolite pairs is a fingerprint of the enzymatic and regulatory reaction networks (Kruger and Ratcliffe, 2012; Toubiana et al., 2013). Furthermore, the results from both pathway and network analyses emphasize the centrality of the phenylpropanoid and flavonoid metabolic pathways in the sorghum responses. Although more sophisticated approaches (e.g., Gaussian graphical models and Bayesian networks; Kim et al., 2011; Kayano et al., 2013) may be needed to decouple direct from indirect variable associations, thus helping the identification of conditionally independent pairwise metabolic relationships, the methodology used in this study comprehensively captured essential features of sorghum defensive metabolism against *C. sublineolum* infection.

### Expression Analyses of Selected Defense-Related Genes in *Sorghum bicolor*

The observed expression of these genes evidently indicates a multiphase defense state in the plants over time. Furthermore, these gene expression results clearly corroborate the information from metabolomics analyses and chemometric models that sorghum responses to *C. sublineolum* infection is time- and cultivar-related. The significant metabolic pathways underlying the response to the fungal infection were found to be the same in all the three cultivars investigated in this study (Figure 4 and Tables 1, 2). However, it is apparent that the kinetics, magnitude, and timing of the responses vary with cultivars: different levels of significant metabolites (expressed as fold change—Table 1) and differential levels and kinetics of gene expression (Figure 8).

Both PAL and PPO are essential enzymes in phenylpropanoid and flavonoid metabolism, leading to the biosynthesis of structural barrier components such as lignin, formation of antimicrobial phenolics such as phytoanticipins and *de novo* biosynthesis of phytoalexins in plant defense events (Mengiste, 2012; Anjum et al., 2013). In the case of the BTT (B) cultivar, the *PAL* gene expression exhibited a bi-phasic pattern, with an initial response at days 3–5, decreasing at day 7, followed by a strong increase at day 9. Similar bi-phasic responses have been reported in plants exhibiting a high level of resistance to pathogen attack (Ding et al., 2011) and in this case might be related to the biotrophic *vs*. necrotrophic stages of the infection. In the case of the SWT (S) cultivar no similar early *PAL* response was observed. For both cultivars, the highest expression of the *PAL* gene was observed from 7 d.p.i. onwards, corresponding to the necrotrophic stage of infection that is accompanied with cell destruction and eventual death. In the case of MHL (M), *PAL* expression levels were very low compared to other two cultivars (Figure 8), suggesting that this cultivar is unable to launch an effective defense response which correlates with the phenotypic observations as described earlier. A clear expression of *PPO* was also observed, with highest levels at 1 d.p.i. in the SWT cultivar, 5 d.p.i. in BTT, and 9 d.p.i. in MHL (Figure 8). Here, no obvious correlation with the type of phytopathogenic interaction could be deduced. However, the substantial expression of both *PAL* and *PPO* genes in sorghum plants responding to *C. sublineolum* clearly correlates and supports the metabolic alterations elucidated by metabolomic analyses, particularly changes and accumulation of phenolic compounds, and including *de novo* biosynthesis of the phytoalexins: apigeninidin and luteolinidin (Figures 4, 5, Tables 1, 2). The general relative low levels of *PAL* and *PPO* (up to 5 d.p.i) expression in the MHL cultivar (Figure 8) corresponds to the low level of observed defense-related phenolics (Table 1, Figure 7). Some previous studies have also reported significant expression levels of both *PPO* and *PAL* in sorghum plants responding to fungal infection, with substantial accumulation and channeling of phenolic compounds to combat the fungal proliferation and colonization (Basavaraju et al., 2009; Anjum et al., 2013).

The two pathogenesis-related genes (*PR3* and *PR10*) were substantially expressed in all cultivars after the pathogen inoculation, with time- and cultivar-dependent expression profiles: early increased expression levels of *PR3* in all cultivars followed by a decrease over the time-course of infection (expect in MHL-cultivar); a Gaussian-type expression profile for *PR10* in both BTT (B) and SWT (S) cultivars, with BTT showing the highest expression levels of *PR10* (at 3–5 d.p.i.) compared to other cultivars, which then decreased (Figure 8). Generally, plants are known to express chitinases (PR3) soon after infection for endolytical hydrolysis of microbial cell walls (Heil, 2002). Studies have shown that in plant: hemibiotrophic pathogen interactions, early defense mechanisms are characterized by a transient induction of *chitinase* (*PR3*) genes with the onset of the biotrophic interaction, which are suppressed with the progression of the infection into the necrotrophic phase (Münch et al., 2008; Vargas et al., 2012). This evidently correlates to the expression profiles of *PR3* in this study—an early significant expression of the gene followed by a remarkable decrease at 5 d.p.i. (Figure 8), which points to an asymptomatic interaction phase. However, the MHL cultivar showed a different profile with a second increase of the *PR3* expression, which could imply a multiphase response or simply other underlying cellular processes. On the other hand, PR10 is a member of a group of intracellular defense-related proteins with ribonuclease-like activity. The PR-10 group is a multigene family having *cis* regulatory elements responsive to various signals like ABA, SA and JA (Sudisha et al., 2012). In general, the PR-10 gene family shows non-specific induction patterns to pathogen and pathogen-derived molecules. These defense-related proteins are widely spread and conserved within the plant kingdom, and are induced following pathogen attack in a wide variety of plant species (Mcgee et al., 2001). Furthermore, the PR10s also exhibit similar amino acid sequence to food and pollen allergens, which point to diverse biological functions (Mcgee et al., 2001; Edreva, 2005). Activation and accumulation of *PR10* gene transcripts have previously also been observed in sorghum infected by *C. sublineolum*, exhibiting increasing expression levels over time (Anjum et al., 2013). Such gradual increase of *PR10* expression levels corresponds to the upregulation patterns observed in this study (Figure 8).

## Conclusion and Perspectives

A systems biology understanding of biochemical and molecular mechanisms which determine the plant immune responses is an essential condition route in the search for new strategies to aid plants to defend themselves against ever-evolving pathogens. Sorghum, one of the most important cereal crops, is greatly threatened by biotic stresses, particularly the hemibiotrophic fungus, *C. sublineolum*. Recent studies have provided insights into key features characterizing sorghum defense responses to *Colletotrichum* infection, ranging from identification of specific defense-related genes (*PAL, PRs, F3H, etc*.), to pinpointing induced resistance events such as production of lignin and accumulation of phenolics. However, a comprehensive description of biochemical and molecular mechanisms that functionally determine and coordinate the events that comprise sorghum's multi-layered immune response is still limited.

The present study, using a LC-MS-untargeted metabolomics approach supported with gene expression analyses, was aimed at obtaining a comprehensive understanding of the defensive metabolism of sorghum in response to *C. sublineolum* inoculation. Multivariate data analysis identified 72 discriminatory/signatory biomarkers of statistical importance. Moreover, the study revealed 23 potential metabolic pathways, with nine being the most significant pathways (phenylalanine metabolism, stilbenoid and gingerol biosynthesis, flavonoid biosynthesis, tryptophan metabolism, riboflavin- and tyrosine metabolism, and phenylpropanoid biosynthesis), and collectively defining the metabolic state of the induced resistance in sorghum. Both metabolic pathway and correlation-based network analyses evidently demonstrated the centrality of the phenylpropanoid and flavonoid pathways in this altered metabolism, involving the modulation and mobilization of phenolic compounds and *de novo* biosynthesis of 3-deoxyanthocyanidin phytoalexins (apigeninidin, luteolinidin), apigenin, luteolin as well as some of the associated conjugates. Furthermore, network analysis revealed some qualitative characteristics of the induced defense response: (i) a high interconnectivity between perturbed metabolites of pathways spanning the defensive metabolism, and (ii) metabolic hubs displaying tight biochemical and structural relationships. These metabolic characteristics suggested coordinated regulatory mechanisms that could be investigated further by future studies.

## Author Contributions

ID and LP conceived the project. ID guided and coordinated the research. FT performed the experimental work. PS the instrumental analysis and AD-T the gene expression analyses. FT analyzed the data and performed the chemometric analyses. All authors contributed to writing and editing of the manuscript. All authors have read and approved the final version of the manuscript.

### Conflict of Interest Statement

The authors declare that the research was conducted in the absence of any commercial or financial relationships that could be construed as a potential conflict of interest.

## References

[B1] Althwab S. Carr T. P. Weller C. L. Dweikat I. M. Schlegel V. (2015). Advances in grain sorghum and its co-products as a human health promoting dietary system. Food Res. Int. 77, 349–359. 10.1016/j.foodres.2015.08.011

[B2] Anjum T. Akram W. Ahmad A. Hussain M. Aslam H. (2013). An insight into the basis of resistance in *Sorghum bicolor* against *Colletotrichum sublineolum*. African J. Microbiol. Res. 7, 1397–1408. 10.5897/AJMR12.1847

[B3] Awika J. M. Rooney L. W. (2004). Sorghum phytochemicals and their potential impact on human health. Phytochemistry 65, 1199–1221. 10.1016/j.phytochem.2004.04.001 15184005

[B4] Balmer D. Flors V. Glauser G. Mauch-Mani B. (2013). Metabolomics of cereals under biotic stress: current knowledge and techniques. Front. Plant Sci. 4, 1–12. 10.3389/fpls.2013.00082 23630531 PMC3632780

[B5] Barupal D. K. Fan S. Fiehn O. (2018). Integrating bioinformatics approaches for a comprehensive interpretation of metabolomics datasets. Curr. Opin. Biotechnol. 54, 1–9. 10.1016/j.copbio.2018.01.010 29413745 PMC6358024

[B6] Basavaraju P. Shetty N. P. Shetty H. S. de Neergaard E. Jørgensen H. J. L. (2009). Infection biology and defence responses in sorghum against *Colletotrichum sublineolum*. J. Appl. Microbiol. 107, 404–415. 10.1111/j.1365-2672.2009.04234.x 19302494

[B7] Boccard J. Rudaz S. (2014). Harnessing the complexity of metabolomic data with chemometrics. J. Chemom. 28, 1–9. 10.1002/cem.2567

[B8] Boddu J. Svabek C. Sekhon R. Gevens A. Nicholson R. L. Jones A. D. (2004). Expression of a putative flavonoid 3′-hydroxylase in sorghum mesocotyls synthesizing 3-deoxyanthocyanidin phytoalexins. Physiol. Mol. Plant Pathol. 65, 101–113. 10.1016/j.pmpp.2004.11.007

[B9] Bolton E. E. Kim S. Bryant S. H. (2011). PubChem3D: similar conformers. J. Cheminform. 3, 1–22. 10.1186/1758-2946-3-13 21554721 PMC3120778

[B10] Bro R. Kjeldahl K. Smilde A. K. Kiers H. A. L. (2008). Cross-validation of component models: a critical look at current methods. Anal. Bioanal. Chem. 390, 1241–1251. 10.1007/s00216-007-1790-1 18214448

[B11] Broadhurst D. Goodacre R. Reinke S. N. Kuligowski J. Wilson I. D. Lewis M. R.. (2018). Guidelines and considerations for the use of system suitability and quality control samples in mass spectrometry assays applied in untargeted clinical metabolomic studies. Metabolomics 14, 1–17. 10.1007/s11306-018-1367-3 29805336 PMC5960010

[B12] Brown M. Wedge D. C. Goodacre R. Kell D. B. Baker P. N. Kenny L. C.. (2011). Automated workflows for accurate mass-based putative metabolite identification in LC/MS-derived metabolomic datasets. Bioinformatics 27, 1108–1112. 10.1093/bioinformatics/btr079 21325300 PMC3709197

[B13] Bylesjö M. Rantalainen M. Cloarec O. Nicholson J. K. Holmes E. Trygg J. (2006). OPLS discriminant analysis: combining the strengths of PLS-DA and SIMCA classification. J. Chemom. 20, 341–351. 10.1002/cem.1006

[B14] Cheynier V. Comte G. Davies K. M. Lattanzio V. Martens S. (2013). Plant phenolics: recent advances on their biosynthesis, genetics, and ecophysiology. Plant Physiol. Biochem. 72, 1–20. 10.1016/j.plaphy.2013.05.009 23774057

[B15] Chong J. Soufan O. Li C. Caraus I. Li S. Bourque G.. (2018). MetaboAnalyst 4.0 : towards more transparent and integrative metabolomics analysis. Nucleic Acids Res. 46, 1–9. 10.1093/nar/gky310 29762782 PMC6030889

[B16] Constabel C. P. Barbehenn R. (2008). Defensive roles of polyphenol oxidase in plants, in Induced Plant Resistance to Herbivory, ed Schaller A. (Victoria, BC, Canada: Springer), 253–269.

[B17] Dicko M. H. Gruppen H. Barro C. Traore A. S. van Berkel W. J. H. Voragen A. G. J. (2005). Impact of phenolic compounds and related enzymes in sorghum varieties for resistance and susceptibility to biotic and abiotic stresses. J. Chem. Ecol. 31, 2671–2688. 10.1007/s10886-005-7619-5 16273434

[B18] Dicko M. H. Gruppen H. Traoré A. S. Voragen A. G. J. van Berkel W. J. H. (2006). Sorghum grain as human food in Africa: relevance of content of starch and amylase activities. African J. Biotechnol. 5, 384–395.

[B19] Ding L. Xu H. Yi H. Yang L. Kong Z. Zhang L.. (2011). Resistance to hemi-biotrophic *F. graminearum* infection is associated with coordinated and ordered expression of diverse defense signaling pathways. PLoS One 6:e19008. 10.1371/journal.pone.0019008 21533105 PMC3080397

[B20] Edreva A. (2005). Pathogenesis-related proteins: research progress in the last 15 years. Gen. Appl. Plant Physiol. 31, 105–124.

[B21] Eriksson L. Trygg J. Wold S. (2008). CV-ANOVA for significance testing of PLS and OPLS® models. J. Chemom. 22, 594–600. 10.1002/cem.1187

[B22] Galindo-Prieto B. Eriksson L. Trygg J. (2015). Variable influence on projection (VIP) for OPLS models and its applicability in multivariate time series analysis. Chemom. Intell. Lab. Syst. 146, 297–304. 10.1016/j.chemolab.2015.05.001

[B23] Godzien J. Alonso-Herranz V. Barbas C. Armitage E. G. (2015). Controlling the quality of metabolomics data: new strategies to get the best out of the QC sample. Metabolomics 11, 518–528. 10.1007/s11306-014-0712-4

[B24] Grapov D. Wanichthanarak K. Fiehn O. (2015). MetaMapR: pathway independent metabolomic network analysis incorporating unknowns. Bioinformatics 31, 2757–2760. 10.1093/bioinformatics/btv194 25847005 PMC4528626

[B25] Heil M. (2002). Induced systemic resistance (ISR) against pathogens in the context of induced plant defences. Ann. Bot. 89, 503–512. 10.1093/aob/mcf076 12099523 PMC4233886

[B26] Ji H. Liu X. S. (2010). Analyzing'omics data using hierarchical models. Nat. Biotechnol. 28, 337–340. 10.1038/nbt.1619 20379180 PMC2904972

[B27] Kang J. Price W. E. Ashton J. Tapsell L. C. Johnson S. (2016). Identification and characterization of phenolic compounds in hydromethanolic extracts of sorghum wholegrains by LC-ESI-MS^n^. Food Chem. 211, 215–226. 10.1016/j.foodchem.2016.05.052 27283625

[B28] Kayano M. Imoto S. Yamaguchi R. Miyano S. (2013). Multi-omics approach for estimating metabolic networks using low-order partial correlations. J. Comput. Biol. 20, 571–582. 10.1089/cmb.2013.0043 23899012

[B29] Kim H. U. Kim T. Y. Lee S. Y. (2011). Framework for network modularization and Bayesian network analysis to investigate the perturbed metabolic network. BMC Syst. Biol. 5(Suppl. 2):S14. 10.1186/1752-0509-5-S2-S14 22784571 PMC3287480

[B30] Kruger N. J. Ratcliffe R. G. (2012). Pathways and fluxes: exploring the plant metabolic network. J. Exp. Bot. 63, 2243–2246. 10.1093/jxb/ers073 22407647

[B31] Lattanzio V. Lattanzio V. M. T. Cardinali A. (2006). Role of phenolics in the resistance mechanisms of plants against fungal pathogens and insects, in Phytochemistry: Advances in Research, ed. Imperto F. (Kerala: Research Signpost), 23–67.

[B32] Liu H. Du Y. Chu H. Shih C. H. Wong Y. W. Wang M.. (2010). Molecular dissection of the pathogen-inducible 3-deoxyanthocyanidin biosynthesis pathway in sorghum. Plant Cell Physiol. 51, 1173–1185. 10.1093/pcp/pcq080 20529887

[B33] Liu W. Saint D. (2002). A new quantitative method of real time reverse transcription polymerase chain reaction assay based on simulation of polymerase chain reaction kinetics. Anal. Biochem. 302, 52–59. 10.1006/abio.2001.5530 11846375

[B34] McDowell J. M. (2013). Genomic and transcriptomic insights into lifestyle transitions of a hemi-biotrophic fungal pathogen. New Phytol. 197, 1032–1034. 10.1111/nph.12141 23373860

[B35] Mcgee J. D. Hamer J. E. Hodges T. K. (2001). Characterization of a PR-10 pathogenesis-related gene family induced in rice during infection with *Magnaporthe grisea*. Mol. Plant-Microbe Interact. 14, 877–886. 10.1094/MPMI.2001.14.7.877 11437261

[B36] Mengiste T. (2012). Plant immunity to necrotrophs. Annu. Rev. Phytopathol. 50, 267–294. 10.1146/annurev-phyto-081211-172955 22726121

[B37] Meyer J. Murray S. L. Berger D. K. (2016). Signals that stop the rot: regulation of secondary metabolite defences in cereals. Physiol. Mol. Plant Pathol. 94, 156–166. 10.1016/j.pmpp.2015.05.011

[B38] Münch S. Lingner U. Floss D. S. Ludwig N. Sauer N. Deising H. B. (2008). The hemibiotrophic lifestyle of *Colletotrichum* species. J. Plant Physiol. 165, 41–51. 10.1016/j.jplph.2007.06.008 17765357

[B39] Nelson P. R. C. Taylor P. A. MacGregor J. F. (1996). Missing data methods in PCA and PLS: score calculations with incomplete observations. Chemom. Intell. Lab. Syst. 35, 45–65. 10.1016/S0169-7439(96)00007-X

[B40] Petrussa E. Braidot E. Zancani M. Peresson C. Bertolini A. Patui S.. (2013). Plant flavonoids—biosynthesis, transport and involvement in stress responses. Int. J. Mol. Sci. 14, 14950–14973. 10.3390/ijms140714950 23867610 PMC3742282

[B41] Poloni A. Schirawski J. (2014). Red card for pathogens: phytoalexins in sorghum and maize. Molecules 19, 9114–9133. 10.3390/molecules19079114 24983861 PMC6271655

[B42] Quideau S. Deffieux D. Douat-casassus C. Pouysegu L. (2011). Plant polyphenols: chemical properties, biological activities, and synthesis. Angew. Chem. Int. Ed. 50, 586–621. 10.1002/anie.201000044 21226137

[B43] Rosato A. Tenori L. Cascante M. De Atauri Carulla P. R. Martins dos Santos V. A. P. Saccenti E. (2018). From correlation to causation: analysis of metabolomics data using systems biology approaches. Metabolomics 14, 1–20. 10.1007/s11306-018-1335-y 29503602 PMC5829120

[B44] Shannon P. Markiel A. Ozier O. Baliga N. S. Wang J. T. Ramage D.. (2003). Cytoscape: a software environment for integrated models of biomolecular interaction networks. Genome Res. 13, 2498–2504. 10.1101/gr.1239303 14597658 PMC403769

[B45] Shigenaga A. Argueso C. (2016). No hormone to rule them all: interactions of plant hormones during the responses of plants to pathogens. Semin. Cell Dev. Biol. 56, 174–189. 10.1016/j.semcdb.2016.06.005 27312082

[B46] Smoot M. E. Ono K. Ruscheinski J. Wang P. L. Ideker T. (2011). Cytoscape 2.8: new features for data integration and network visualization. Bioinformatics 27, 431–432. 10.1093/bioinformatics/btq675 21149340 PMC3031041

[B47] Sudisha J. Sharathchandra R. G. Amruthesh K. N. Kumar A. Shetty H. S. (2012). Pathogenesis related proteins in plant defense response, in Plant Defence: Biological Control (Dordrecht: Springer Netherlands), 379–403. 10.1007/978-94-007-1933-0_17

[B48] Sumner L. W. Amberg A. Barrett D. Beale M. H. Beger R. Daykin C. A.. (2007). Proposed minimum reporting standards for chemical analysis. Metabolomics 3, 211–221. 10.1007/s11306-007-0082-2 24039616 PMC3772505

[B49] Szekely G. J. Rizzo M. L. (2005). Hierarchical clustering via Join between-within distances: extending Ward's minimum variance method. J. Classif. 22, 151–183. 10.1007/s00357-005-0012-9

[B50] Tesso T. Perumal R. Little C. R. Adeyanju A. Radwan G. L. Prom L. K. (2012). Sorghum pathology and biotechnology - a fungal disease perspective: part II. anthracnose, stalk rot, and downy mildew. Eur. J. Plant Sci. Biotechnol. 6, 31–44.

[B51] Toubiana D. Fernie A. R. Nikoloski Z. Fait A. (2013). Network analysis: tackling complex data to study plant metabolism. Trends Biotechnol. 31, 29–36. 10.1016/j.tibtech.2012.10.011 23245943

[B52] Trygg J. Holmes E. Lundstedt T. (2007). Chemometrics in metabonomics. J. Proteome Res. 6, 469–479. 10.1021/pr060594q 17269704

[B53] Tugizimana F. Piater L. A. Dubery I. A. (2013). Plant metabolomics : a new frontier in phytochemical analysis. S. Afr. J. Sci. 109, 18–20. 10.1590/sajs.2013/20120005

[B54] Tugizimana F. Steenkamp P. Piater L. Dubery I. (2016). A conversation on data mining strategies in LC-MS untargeted metabolomics: pre-processing and pre-treatment steps. Metabolites 6, 1–18. 10.3390/metabo6040040 27827887 PMC5192446

[B55] Vargas W. A. Martin J. M. S. Rech G. E. Rivera L. P. Benito E. P. Diaz-Minguez J. M.. (2012). Plant defense mechanisms are activated during biotrophic and necrotrophic development of *Colletotricum graminicol*a in maize. Plant Physiol. 158, 1342–1358. 10.1104/pp.111.190397 22247271 PMC3291271

[B56] Wiklund S. Johansson E. Sjöström L. Mellerowicz E. J. Edlund U. Shockcor J. P.. (2008). Visualization of GC/TOF-MS-based metabolomics data for identification of biochemically interesting compounds using OPLS class models. Anal. Chem. 80, 115–122. 10.1021/ac0713510 18027910

[B57] Wu G. Johnson S. K. Bornman J. F. Bennett S. J. Fang Z. (2017). Changes in whole grain polyphenols and antioxidant activity of six sorghum genotypes under different irrigation treatments. Food Chem. 214, 199–207. 10.1016/j.foodchem.2016.07.089 27507466

[B58] Xia J. Sinelnikov I. V. Han B. Wishart D. S. (2015). MetaboAnalyst 3.0–making metabolomics more meaningful. Nucleic Acids Res. 43, W251–W257. 10.1093/nar/gkv380 25897128 PMC4489235

