# Supplementary material for: Metabolomic Analysis of Defense-Related Reprogramming in Sorghum bicolor in Response to Colletotrichum sublineolum Infection Reveals a Functional Metabolic Web of Phenylpropanoid and Flavonoid Pathways
Source: Front Plant Sci. 2019 Jan 4;9:1840. doi: 10.3389/fpls.2018.01840 (PMC6328496; doi:10.3389/fpls.2018.01840)
Supplement: Supplementary file 1 [file Data_Sheet_1.PDF]

Supplementary Material

Metabolomic analysis of defence-related reprogramming in *Sorghum bicolor* in response to *Colletotrichum sublineolum* infection reveals a functional metabolic web of phenylpropanoid and flavonoid pathways

Fidele Tugizimana, Arnaud T. Djami-Tchatchou, Paul A. Steenkamp, Lizelle A. Piater, Ian A. Dubery*

Research Centre for Plant Metabolomics, Department of Biochemistry, University of Johannesburg, Auckland Park, Johannesburg, South Africa

*** Correspondence:** Ian A. Dubery; idubery@uj.ac.za

1 ADDITIONAL BACKGROUND ON THE *Sorghum bicolor : Colletotrichum sublineolum* PHYTOPATHOSYSTEM

In general, as with all plants, sorghum is equipped with a non-specific, passive protective system constituted by structural and chemical barriers that prevent or attenuate invasion by potential biotic stressors that threaten its physiology. However, when these barriers are breached and following the perception of *C. sublineolum* intrusion, sorghum deploys an array of defence mechanisms to counter the infection. Among these currently known from sorghum infected by *C. sublineolum* are formation of papillae, the production of reactive oxygen species (ROS), induction of defence-related genes that encode phenylalanine ammonia-lyase (PAL) and polyphenol oxidases (PPO), the synthesis and accumulation of proteinase inhibitors and pathogenesis-related (PR)-proteins (such as chitinases and ß-glucanases), and changes in polyphenolics and synthesis of phytoalexins (Balmer et al., 2013; Basavaraju et al., 2009; Poloni and Schirawski, 2014). ROS production, specifically H_2O_2, is one of the earliest responses, and involved in defence reactions such as cell wall strengthening, the hypersensitive response (HR) reaction as well as in signal transduction (Mittler *et al.*, 2011; Vargas *et al.*, 2012; Lehmann *et al.*, 2015). However, recent studies have demonstrated that in hemibiotrophic interactions, ROS (particularly H_2O_2) have different roles: being part of defensive reactions during the biotrophic phase, whereas it is involved in pathogenesis during the necrotrophic phase (Mayer et al., 2001; Basavaraju et al., 2009).

Furthermore, these sorghum defence responses to *C. sublineolum*, comprising induction of PAL and PPO, implies defence-related changes at the metabolome level – as the latter reflects the dynamic equilibrium of intertwined networks of enzymatic and non-enzymatic biochemical processes that define the cellular and organismal metabolism, that characterises the defensive physiological state of the plant (Basavaraju et al., 2009; McKnight, 2010; Ray, 2010; Vargas et al., 2012). Most studies have demonstrated that the metabolic changes related to sorghum defence responses to fungal infection involve the biosynthesis of a unique and distinct class of flavonoid phytoalexins, the 3-

deoxyanthocynidins, namely apigeninidin and luteolinidin (**Figures S1-S2**) (Ahuja et al., 2012; Balmer et al., 2013; Poloni and Schirawski, 2014). These are known to be synthesised in the cytoplasm of infected epidermal sorghum cells and accumulate in intracellular (initially colourless) inclusion bodies, which migrate towards the site of fungal penetration, releasing activated compounds that kill both the fungus and the cells where synthesised (Basavaraju et al., 2009; Liu et al., 2010; Poloni and Schirawski, 2014).

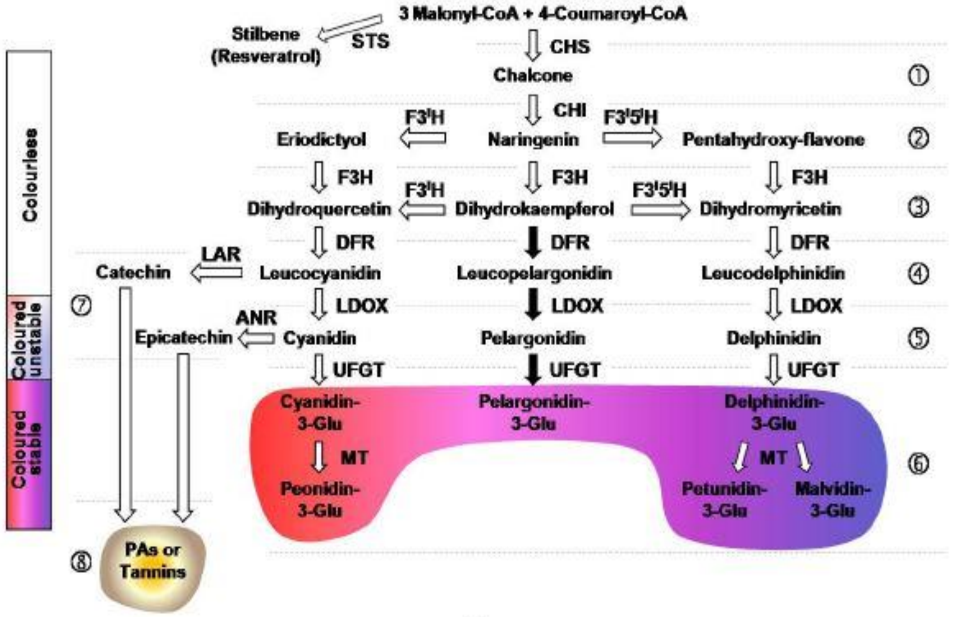

(A)

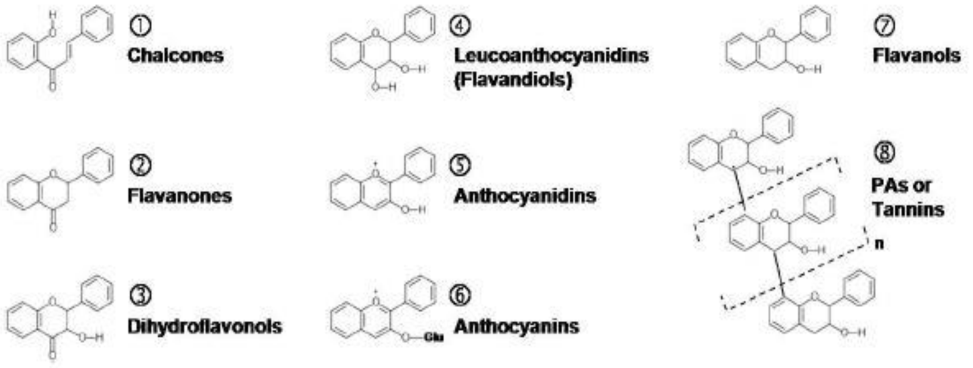

(B)

Figure S1. (**A**) **Global scheme of the flavonoid biosynthetic pathway in plant cells**. Anthocyanins are synthesised by a multienzyme complex loosely associated to the endoplasmic reticulum (CHS, chalcone synthase; CHI, chalcone isomerase; F3H, flavanone 3-hydroxylase; F3'H, flavonoid 3'-hydroxylase; F3'5'H, flavonoid 3',5'-hydroxylase; DFR, dihydroflavonol reductase; LDOX, leucoanthocyanidin oxidase; UFGT, UDP-glucose flavonoid 3-O-glucosyl transferase; MT, methyltransferase). Proanthocyanidins (PAs) synthesis branches off the anthocyanin pathway (LAR, leucoanthocyanidin reductase; ANR, anthocyanidin reductase; STS, stilbene synthase); the black arrows refer to biosynthetic steps missing in grapevine. Numbers next to the flavonoid groups are related to the chemical structures of the major flavonoid groups shown in (**B**) (Petrussa et al., 2013).

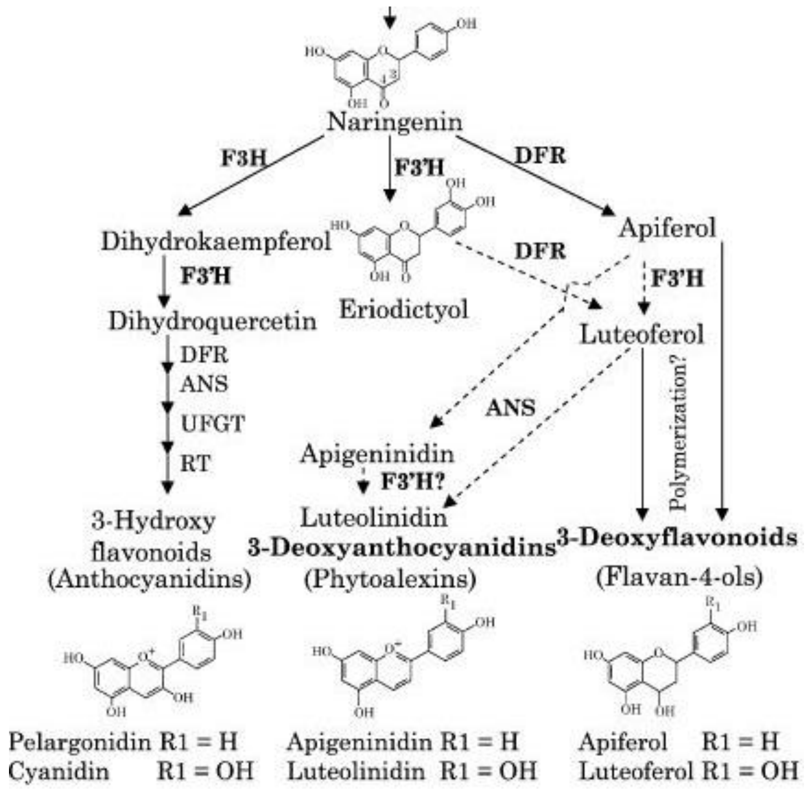

Figure S2. Schematic representation of the flavonoid biosynthesis pathway in maize and sorghum. Shown are three branches diverging from naringenin and leading to the synthesis of 3-hydroxyflavonoids (anthocyanins), 3-deoxyflavonoids (flavan-4-ols/phlobaphenes) and 3-deoxyanthocyanidins (phytoalexins). Solid arrows indicate the steps where the respective gene has been isolated and characterised. Dotted arrows indicate the proposed or uncharacterised steps. Enzymes shown are: PAL, phenylalanine ammonia-lyase; C4H, cinnamate 4-hydroxylase; 4CL, coumarate 4-ligase; CHS, chalcone synthase; CHI, chalcone isomerase; F3H, flavanone 3-hydroxylase; F3'H, flavonoid 30-hydroxylase; DFR, dihydroflavonol reductase; UFGT, UDPG-flavonoid glucosyl transferase; RT, rhamnosyl transferase, and ANS, anthocyanidin synthase (Boddu et al., 2004). Apigeninidin, luteolinidin and associated derivatives are important chemical defences in sorghum.

The 3-deoxyanthocyanidin molecules (orange-red colouration, **Figure S1**) are structurally similar to anthocyanins except for the absence of C-3 hydroxylation. The biosynthesis of these phytoalexins after fungal infection occurs through the coordinated expression of genes encoding enzymes of sequential biochemical reactions. These enzymes include, among others, PAL – the linking step between primary and secondary metabolism – chalcone synthase (CHS), cinnamate 4-hydoxylase (C4H), chalcone isomerase (CHI), dihydroflavonol 4-reductase (DFR) and flavonoid 3'-hydroxylase (F3'H) (**Figures S1-S2**) (Boddu et al., 2004; Shih et al., 2006; Poloni and Schirawski, 2014; Mizuno et al., 2016). Although the final steps of the pathogen-induced biosynthesis of these antifungal compounds are not fully elucidated, enzymes such as anthocyanidin synthase (ANS) and F3'H are reported to be essential in the production of these 3-deoxyanthocyanidin compounds.

2 GENE EXPRESSION ANALYSES

Table S1. Base composition of primers designed for the selected genes investigated using qPCR.

Genes	Direction	Primer sequence (5’ to 3’)	Tm (˚C)
PR3	Forward	TGGAACCGCTTCTACGATGT	60
	Reverse	CGCGCGCTCTTATTTTATCT	
F3’H	Forward	CGACGACATGATGAACGG	60
	Reverse	CGATCGATTGCTGTTCCC	
PR10	Forward	CCGACGCCTACAACTAAATCTG	60
	Reverse	CATACACCACACACCGCATAGAG	
PAL	Forward	ATGAAGCACGCCAAGAAG	60
	Reverse	CGGAGGACCTCGATCTG	
PPO	Forward	GATTCTTGGGAAGCTCATCG	60
	Reverse	GTCTTGTTGGCGTAGATCG	
EF1α	Forward	TATCCTCCTCTTGGTCGCTTTG	60
	Reverse	GGGTCCTTCTTCTCCACGCTCT	
UBC18	Forward	AAAGCAACGCCCAGCCGATAA	60
	Reverse	GGACAATCAGGAAACCCATCAC	

3 EVALUATION OF ANTHRACNOSE SYMPTOM DEVELOPMENT – SYMPTOMATOLOGY

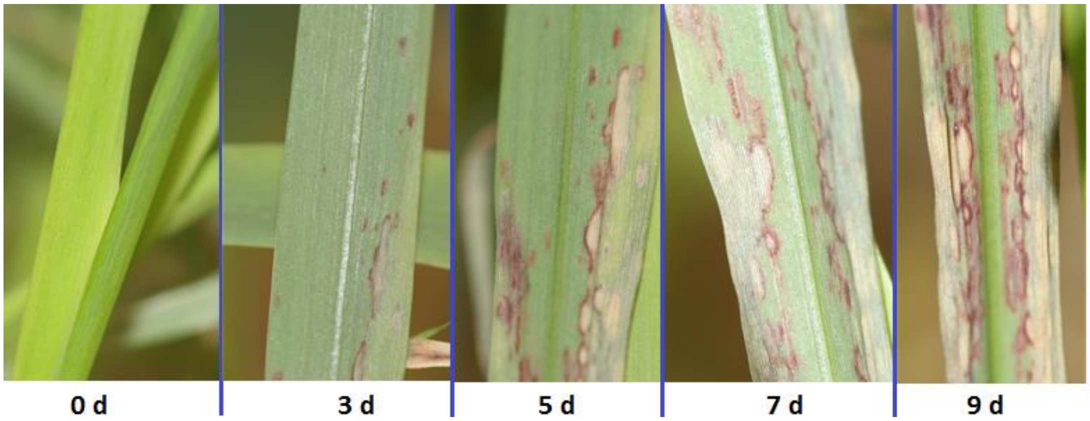

Figure S3. Typical development of anthracnose symptoms on sorgum leaves in response to *C. sublineolum* infection. Following inoculation with fungal spores, development of lesions on the sorghum leaves (***e.g.* NS 5511 / BTT cultivar)**: were observed over time. Lesions started appearing from 3 d.p.i. and spread progressively over time. At 9 d.p.i. the lesions appear to cover almost the whole leaf surface. These symptom development patterns were comparable in both SWT and BTT cultivars, but the MHL cultivar showed quicker development of symptoms and was severely affected by the pathogen infection (**Table S2**).

Table S2. Disease severity-rating for sorghum cultivars (MHL, SWT and BTT) responding to *C. sublineolum* infection over a period of 9 days post-inoculation.

Days (d.p.i.)	Disease severity (1-5)*		
	MHL	SWT	BTT
1	1	1	1
2	2	1	1
3	2-3	2	2
4	3	2-3	2
5	3-4	3	3
6	4	3-4	3
7	4-5	3-4	3-4
8	5	4-5	4
9	5	4-5	4-5

***Disease severity-index scoring for symptom development**.
1 = no visible symptoms;
2 = 1-25% lesions/spots and plant starting to wilt;
3 = 25-50% lesions and 25% of plant wilted;
4 = 50-75% lesions and 50% of plant wilted;

5 = 75-100% lesions and >50% of plant wilted.

As the fungal infection-related cellular structures sequentially develop, the pathogen is initially non-destructive, manoeuvring the host physiology to maintain viability of infected tissue and relying on the nutritional materials from live host cells, and forming more specialised cells to spread infectiously. On the plant side, this biotrophic phase is asymptomatic and short lived, lasting for 2-3 days after inoculation (Liu et al., 2010; Mengiste, 2012; Tesso et al., 2012; Vargas et al., 2012). At about 3 d.p.i.,

circular and elliptical spots start appearing on the sorghum infected-leaves. Visual inspection of the development of symptoms over time showed a gradual progression of the disease, with leaves being almost completely covered by lesions on the 9th day post-infection (**Figure S3** and **Table S2**). This symptomatic phase corresponds to the fungus having switched to the necrotrophic lifestyle – destructively affecting the host physiology and killing (locally) the host cells and tissue, and aggressively invading and colonising other parts of the leaf and subsequently all aerial parts of the plant.

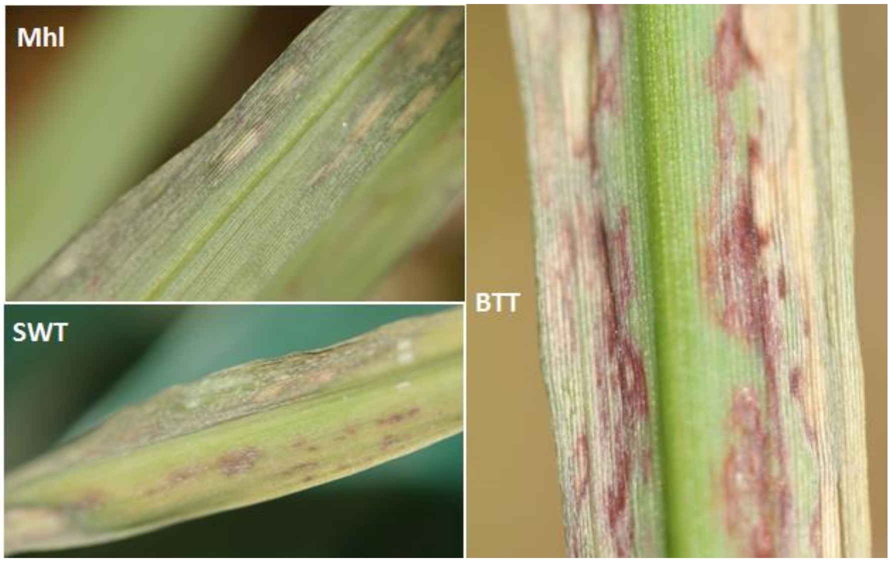

Figure S4. Differential symptoms expression in the three sorghum cultivars – MHL, SWT and BTT. The development of the symptoms in the three sorghum cultivars visually show some cultivar-related differences. As indicated in **Table S2**, the MHL cultivar appeared to be severely affected by the fungal infection, and the symptoms developed show differences compared to the SWT and BTT cultivars: *e.g.* less reddish colouring around the lesions. Both SWT and BTT cultivars show similar symptom appearances and development as seen in these selected pictures.

Responding to the fungal attack, the immune responses of sorghum are activated, involving complex cellular, histological, biochemical and molecular processes that, together, limit disease symptom expression or kill and limit pathogen proliferation. Some of these immune response events include programmed cell death of infected host cells, production of various defence-related secondary metabolites such as phenolics and antifungal phytoalexins (Boddu et al., 2004; Liu et al., 2010; Mengiste, 2012; Poloni and Schirawski, 2014); hence the (reddish and dark) colouring at the symptomatic lesions (**Figures S3-S4**). However, symptomatically, the sorghum–*C. sublineolum* interactions show some differential nuances comparing the three sorghum cultivars (**Figure S4** and **Table S2**). The SWT and BTT cultivars showed similar development of symptom, with the BTT cultivar having more reddish elongated spots than those observed on the SWT leaves. However, the latter wilted more quickly than BTT plants (**Table S2**). The phenotypic observations can be interpreted as BTT exhibiting a stronger defence response than SWT, with possible HR lesions and associated with phytoalexin accumulation, leading to the purple colour of the surrounding tissue. In contrast, the development of lesions on MHL cultivar was characterised by dark green areas on the leaf, zones of

leaf tissue dying and the wilting of the whole leaf, as the infection progresses. Minimal reddish spots were visible on the leaves of the MHL cultivar. The MHL plants wilted and showed signs of dying before the other two cultivars (**Figure S4** and **Table S2**). Here, the phenotype response can be interpreted as the cultivar having little or no resistance against *C. sublineolum*.

4 METABOLOMIC ANALYSES

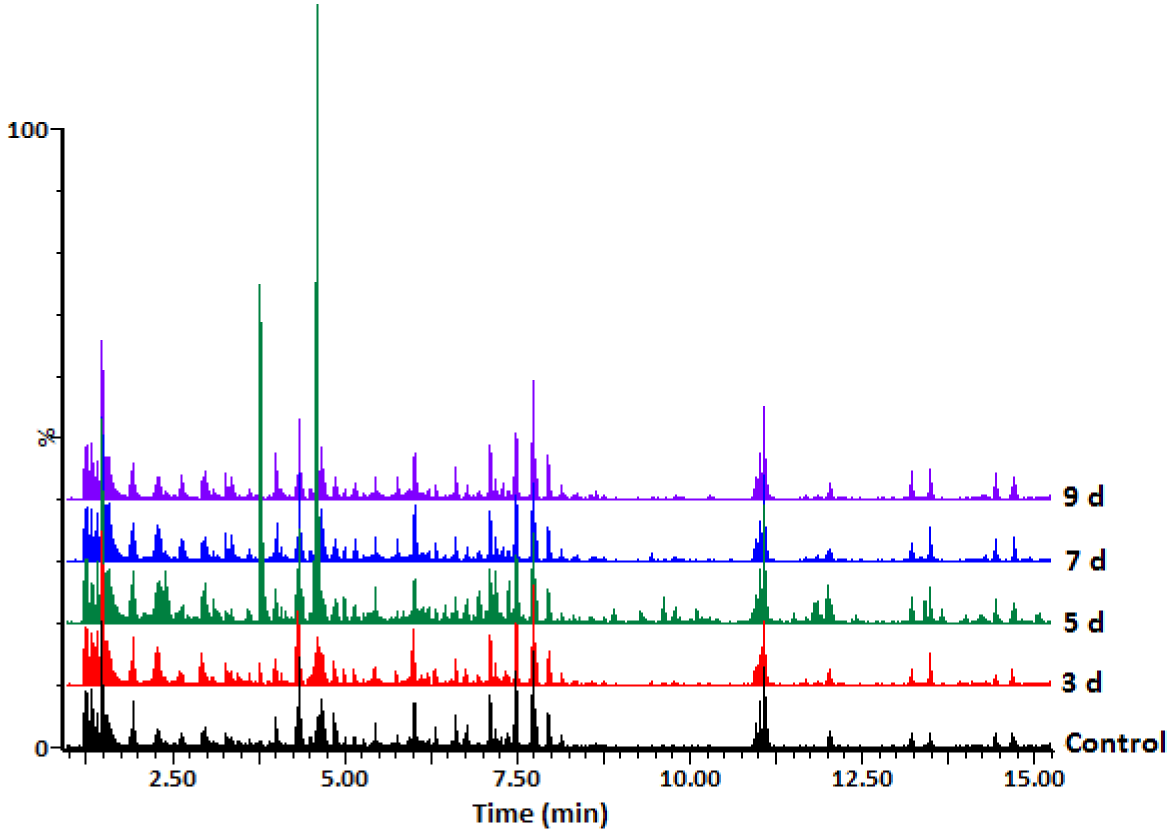

Figure S5A. UHPLC-MS BPI chromatograms (ESI positive data): typical mass chromatograms of the samples from sorghum NS 5511 / BTT cv, responding to fungal infection. Control refers to samples from non-infected plants. The 3 – 9 d.p.i. samples are also indicated. Visual inspection of these mass chromatograms show resolution of a number of ion peaks, reflecting the complexity of the extracts. Most of the negatively ionised compounds were mid-polar. Furthermore, differential peak populations can be observed on the mass chromatograms, pointing to infection-related metabolic changes.

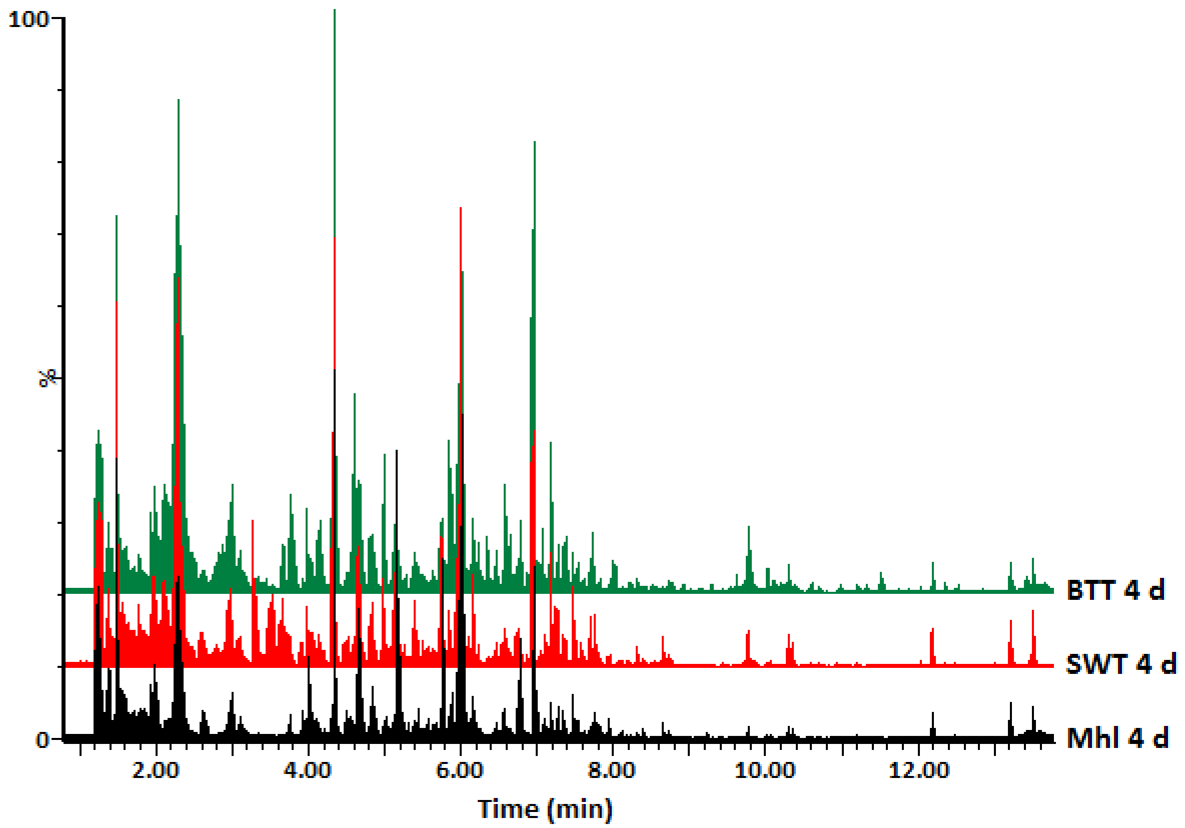

Figure S5B. UHPLC-MS BPI chromatograms (ESI negative data): typical mass chromatograms of the samples from the three sorghum cvs used in this study (abbreviated, MHL, SWT and BTT). Chromatographically comparing the three cvs, using mass chromatograms of treated samples – 4 d.p.i., shows that there are cultivar-related differences with regard to peak populations and peak intensities. This points to cv-related metabolic differences, as pointed out by chemometric analyses in the main text of this study.

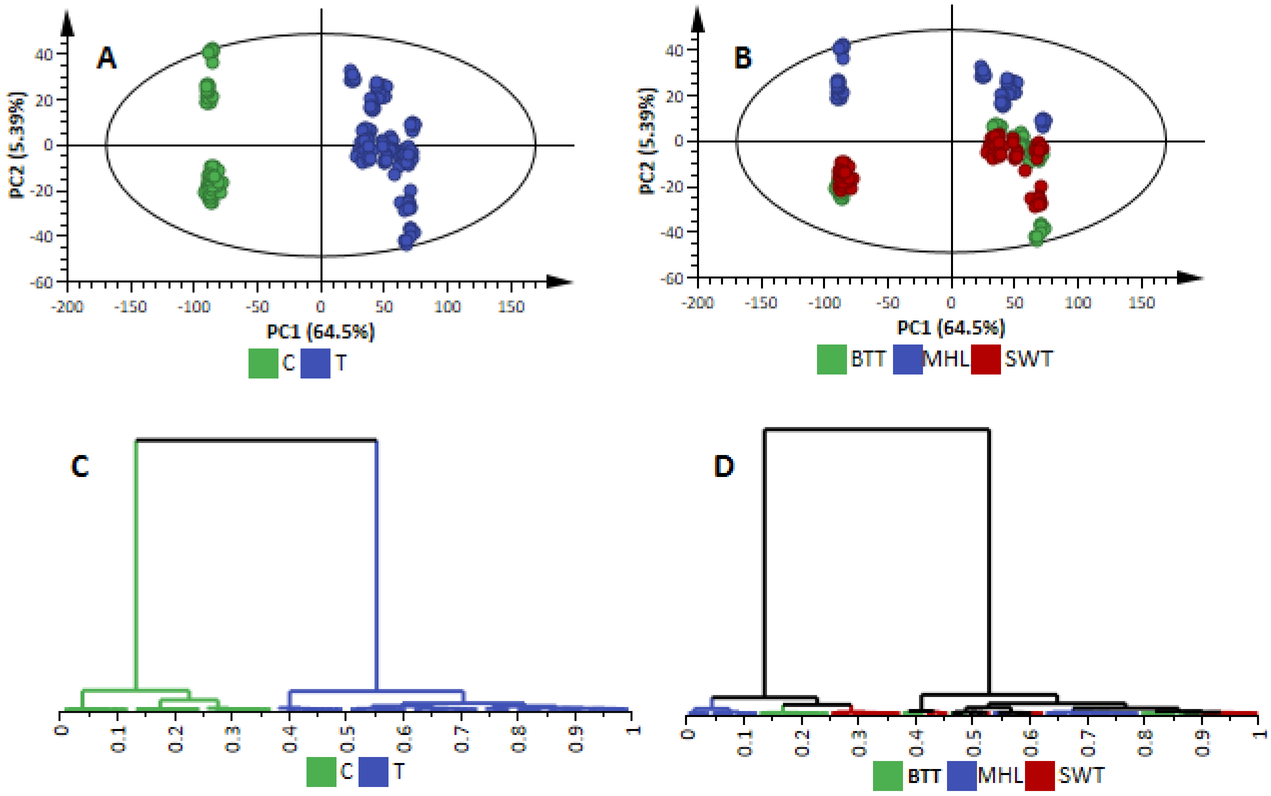

Figure S6. Unsupervised chemometric modelling (ESI positive data): (**A**) A PCA scores scatter plot of all the samples (MHL, SWT and BTT), coloured according to the treatment (control (green) *vs*. infected (blue)). (**B**) The score plot in (**A**) but coloured according to cultivars (BTT = green, MHL= blue, SWT = brown). The PCA model presented here was an 18-component model (of the Pareto-scaled data matrix **X**), with R^2 of 0.902 and Q^2 of 0.820. (**C**) and (**D**) are HCA dendrograms corresponding to (**A**) and (**B**), respectively. The unsupervised modelling provided a global overview of the data as shown in the PCA scores plots and HCA dendrograms, allowing the identification of sample grouping and natural clustering in multivariate space: treatment-related and cultivar-dependent grouping were observed (**A-D**).

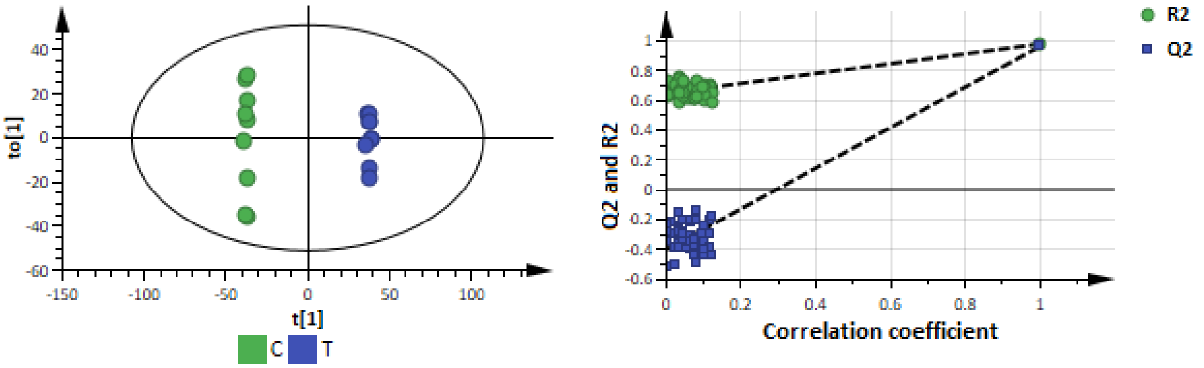

Figure S7. OPLS-DA modelling and variable/feature selection. (**A**) A typical scores scatter plot of the OPLS-DA model (ESI negative data) separating 'control (C -green) *vs*. infected plants (T - blue)' at 7 d.p.i. (1 + 1 + 0 components, $R^2X = 0.611$, $Q^2 = 0.994$, CV-ANOVA *p*-value = 2.4×10^{-14}). In the scores space, the two groups (control–C and treated–T) are clearly separated. (**B**) The **response permutation test plot** ($n = 50$) for the same OPLS-DA model: R^2 (0.0, 0.335) and Q^2 (0.0, − 0.403) values of the permuted models are represented on the left-hand side of the plot, corresponding to *y*-axis intercepts.

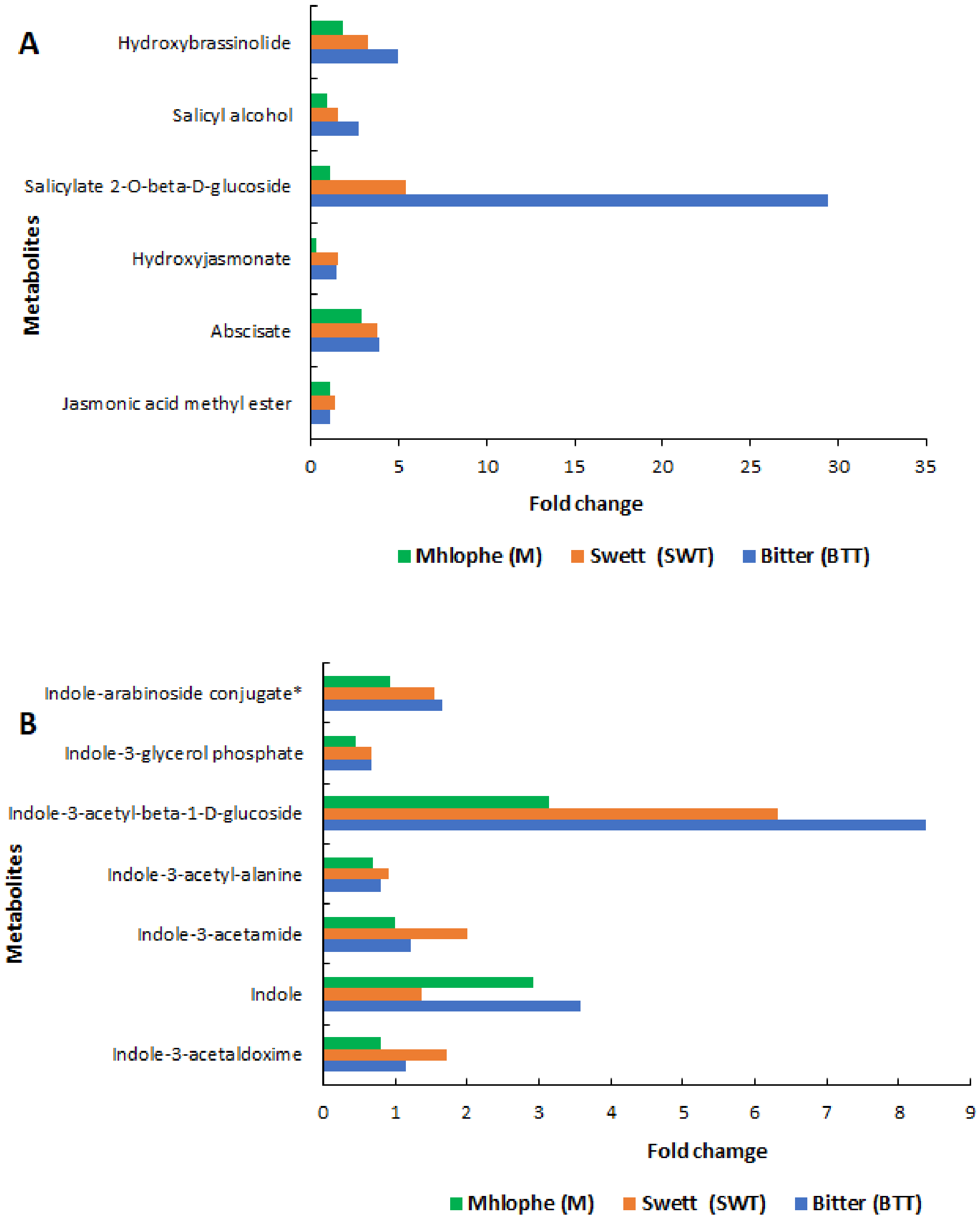

Figure S8. **Relative quantification of selected metabolites**: (**A**) phytohormones and (**B**) indole compounds. The relative level of each metabolite is expressed as fold change: data from treated- *vs.* control samples, *i.e.* infected plants (at 5 d.p.i.) *vs.* control (non-infected) plants (**Table 1**). These annotated (MI-level 2) discriminating metabolites were selected based on OPLS-DA S-plots, all with *p*-values < 0.05 and VIP scores > 1.0. The graph displays infographically the cultivar-dependent quantitative differences in the phytohormones and indole compounds in response to *C. sublineolum* infection.

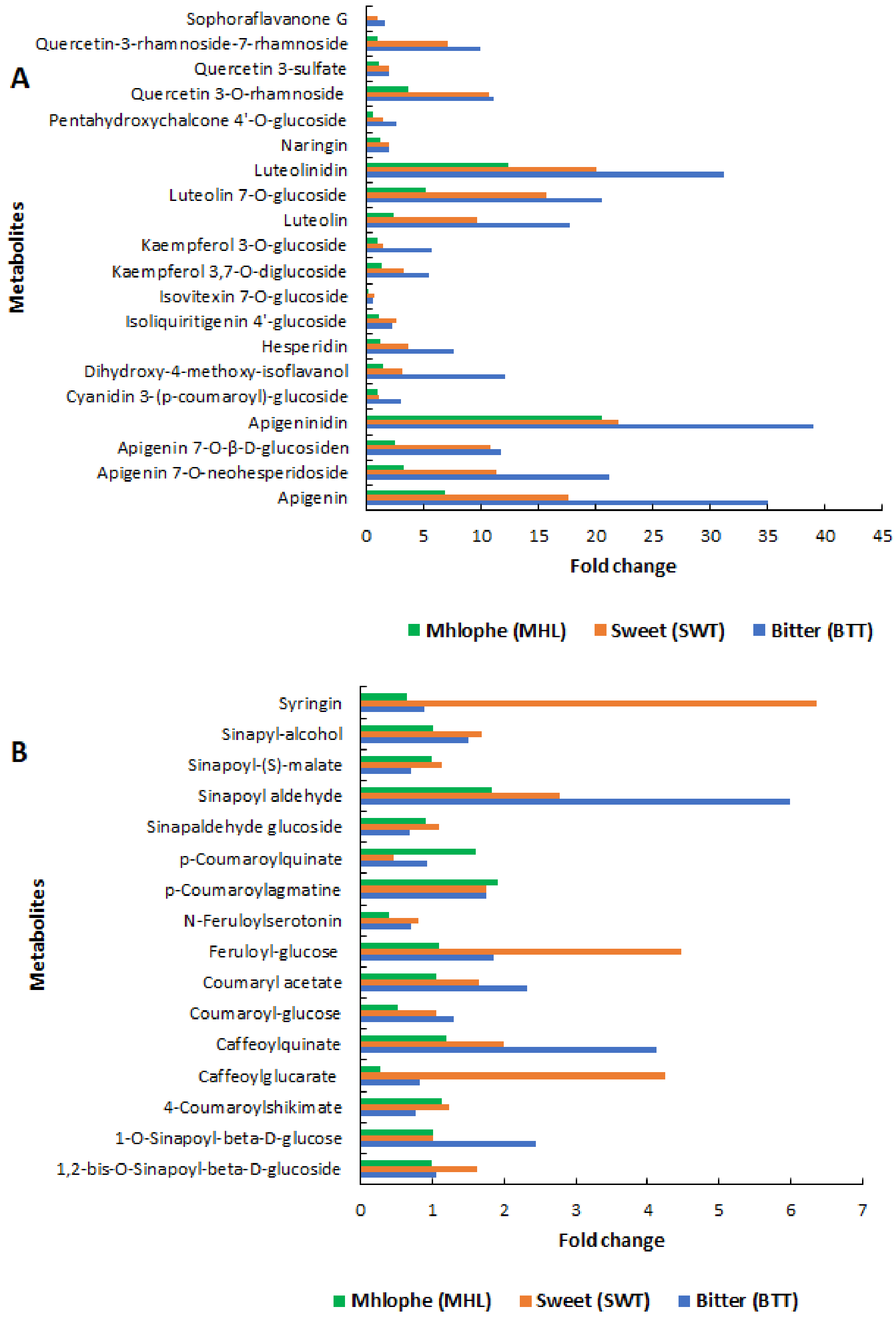

Figure S9. **Relative quantification of selected metabolites**: (**A**) flavonoids and (**B**) hydroxycinnamic acid derivatives. The relative level of each metabolite is expressed as fold change: data from treated- *vs.* control samples, *i.e.* infected plants (at 5 d.p.i.) *vs.* control (non-infected) plants (**Table 1**). These annotated (MI-level 2) discriminating metabolites were selected based on OPLS-DA S-plots, all with *p*-values < 0.05 and VIP scores > 1.0. The graph displays infographically the cultivar-dependent quantitative differences in the flavonoids and hydroxycinnamic acid derivatives in response to *C. sublineolum* infection.

REFERENCES

Ahuja, I., Kissen, R., and Bones, A. M. (2012). Phytoalexins in defense against pathogens. *Trends Plant Sci.* 17, 73–90. doi:10.1016/j.tplants.2011.11.002.

Balmer, D., Flors, V., Glauser, G., and Mauch-Mani, B. (2013). Metabolomics of cereals under biotic stress: current knowledge and techniques. *Front. Plant Sci.* 4, 1–12. doi:10.3389/fpls.2013.00082.

Basavaraju, P., Shetty, N. P., Shetty, H. S., de Neergaard, E., and Jørgensen, H. J. L. (2009). Infection biology and defence responses in sorghum against *Colletotrichum sublineolum*. *J. Appl. Microbiol.* 107, 404–415.

Boddu, J., Svabek, C., Sekhon, R., Gevens, A., Nicholson, R. L., Jones, A. D., et al. (2004). Expression of a putative *flavonoid 3′-hydroxylase* in sorghum mesocotyls synthesizing 3-deoxyanthocyanidin phytoalexins. *Physiol. Mol. Plant Pathol.* 65, 101–113. doi:10.1016/j.pmpp.2004.11.007.

Lehmann, S., Serrano, M., L'Haridon, F., Tjamos, S. E., and Metraux, J.-P. (2015). Reactive oxygen species and plant resistance to fungal pathogens. *Phytochemistry* 112, 54–62. doi:10.1016/j.phytochem.2014.08.027.

Liu, H., Du, Y., Chu, H., Shih, C. H., Wong, Y. W., Wang, M., et al. (2010). Molecular dissection of the pathogen-inducible 3-deoxyanthocyanidin biosynthesis pathway in sorghum. *Plant Cell Physiol.* 51, 1173–1185. doi:10.1093/pcp/pcq080.

Mayer, A. M., Staples, R. C., and Gil-ad, N. L. (2001). Mechanisms of survival of necrotrophic fungal plant pathogens in hosts expressing the hypersensitive response. *Phytochemistry* 58, 33–41.

McKnight, S. L. (2010). On getting there from here. *Science.* 330, 1338–1339. doi:10.1126/science.1199908.

Mengiste, T. (2012). Plant immunity to necrotrophs. *Annu. Rev. Phytopathol.* 50, 267–294. doi:10.1146/annurev-phyto-081211-172955.

Mittler, R., Vanderauwera, S., Suzuki, N., Miller, G., Tognetti, V. B., Vandepoele, K., et al. (2011). ROS signaling: the new wave? *Trends Plant Sci.* 16, 300–9. doi:10.1016/j.tplants.2011.03.007.

Mizuno, H., Yazawa, T., Kasuga, S., Sawada, Y., Kanamori, H., Ogo, Y., et al. (2016). Expression of Flavone Synthase II and Flavonoid 3′-Hydroxylase is associated with color variation in tan-colored injured leaves of Sorghum. *Front. Plant Sci.* 7, 1–10. doi:10.3389/fpls.2016.01718.

Petrussa, E., Braidot, E., Zancani, M., Peresson, C., Bertolini, A., Patui, S., et al. (2013). Plant flavonoids—biosynthesis, transport and involvement in stress responses. *Int. J. Mol. Sci.* 14, 14950–14973. doi:10.3390/ijms140714950.

Poloni, A., and Schirawski, J. (2014). Red card for pathogens: phytoalexins in sorghum and maize. *Molecules* 19, 9114–9133. doi:10.3390/molecules19079114.

Ray, L. B. (2010). Metabolism is not boring. *Science.* 330, 1337–1337. doi:10.1126/science.330.6009.1337.

Shih, C.-H., Chu, I. K., Yip, W. K., and Lo, C. (2006). Differential expression of two flavonoid 3'-hydroxylase cDNAs involved in biosynthesis of anthocyanin pigments and 3-deoxyanthocyanidin phytoalexins in sorghum. *Plant Cell Physiol.* 47, 1412–1419. doi:10.1093/pcp/pcl003.

Tesso, T., Perumal, R., Little, C. R., Adeyanju, A., Radwan, G. L., Prom, L. K., et al. (2012). Sorghum pathology and biotechnology - a fungal disease perspective: part II. anthracnose, stalk rot, and downy mildew. *Eur. J. Plant Sci. Biotechnol.* 6, 31–44.

Vargas, W. A., Martin, J. M. S., Rech, G. E., Rivera, L. P., Benito, E. P., Diaz-Minguez, J. M., et al. (2012). Plant defense mechanisms are activated during biotrophic and necrotrophic development of *Colletotricum graminicola* in maize. *Plant Physiol.* 158, 1342–1358. doi:10.1104/pp.111.190397.
